# Intelligent Vascularized 3D/4D/5D/6D-Printed Tissue Scaffolds

**DOI:** 10.1007/s40820-023-01187-2

**Published:** 2023-10-31

**Authors:** Xiaoyu Han, Qimanguli Saiding, Xiaolu Cai, Yi Xiao, Peng Wang, Zhengwei Cai, Xuan Gong, Weiming Gong, Xingcai Zhang, Wenguo Cui

**Affiliations:** 1 Department of Orthopaedics, Shanghai Key Laboratory for Prevention and Treatment of Bone and Joint Diseases, Shanghai Institute of Traumatology and Orthopaedics, Ruijin Hospital, Shanghai Jiao Tong University School of Medicine, 197 Ruijin 2nd Road, Shanghai, 200025 People’s Republic of China; 2 Department of Orthopedics, Jinan Central Hospital, Shandong First Medical University and Shandong Academy of Medical Sciences, 105 Jiefang Road, Lixia District, Jinan, 250013 Shandong People’s Republic of China; 3 Department of Biomedical Engineering, College of Life Science and Technology, Huazhong University of Science and Technology, Wuhan, 430074 Hubei People’s Republic of China; 4 School of Engineering and Applied Sciences, Harvard University, Cambridge, MA 02138 USA; 5 University of Texas Southwestern Medical Center, Dallas, TX 75390-9096 USA

**Keywords:** Intelligent, Additive manufacturing, Tissue engineering, Vascularization, Osteogenesis

## Abstract

Blood vessels are essential for nutrient and oxygen delivery and waste removal. Scaffold-repairing materials with functional vascular networks are widely used in bone tissue engineering. Additive manufacturing is a manufacturing technology that creates three-dimensional solids by stacking substances layer by layer, mainly including but not limited to 3D printing, but also 4D printing, 5D printing and 6D printing. It can be effectively combined with vascularization to meet the needs of vascularized tissue scaffolds by precisely tuning the mechanical structure and biological properties of smart vascular scaffolds. Herein, the development of neovascularization to vascularization to bone tissue engineering is systematically discussed in terms of the importance of vascularization to the tissue. Additionally, the research progress and future prospects of vascularized 3D printed scaffold materials are highlighted and presented in four categories: functional vascularized 3D printed scaffolds, cell-based vascularized 3D printed scaffolds, vascularized 3D printed scaffolds loaded with specific carriers and bionic vascularized 3D printed scaffolds. Finally, a brief review of vascularized additive manufacturing-tissue scaffolds in related tissues such as the vascular tissue engineering, cardiovascular system, skeletal muscle, soft tissue and a discussion of the challenges and development efforts leading to significant advances in intelligent vascularized tissue regeneration is presented.

## Highlights

- Comprehensive and systematic discussion of vascularized additive manufacturing scaffolds for bone tissue repair is provided.
- The development mechanism of blood vessels and the relationship between bone tissue engineering and blood vessels are discussed.
- Vascularized additively manufactured scaffolds in tissue repair are discussed in terms of issues, opportunities, and challenges.
- Intelligent vascularized 3D/4D/5D/6D-printed tissue scaffolds are discussed.

## Graphical abstract

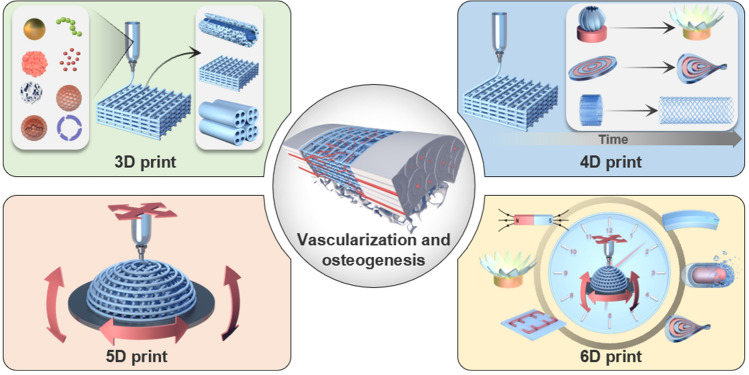

## Introduction

Professor Charles Hull first proposed the concept of 3-dimensional printing (3D printing) in the 1980s and invented the first 3D printer, thus establishing the 3D printing system [1, 2]. In 3D printing technology, also known as additive manufacturing, a 3D model is first created through computer-aided design (CAD) software and then printed in a predefined path using a robotic arm or nozzle. Recently, 3D bioprinting has emerged as a promising alternative method and is now widely used to manufacture functional tissue structures with complex geometries [3–5]. This technology aims to replace or regenerate damaged tissues and organs, such as bone, liver, cartilage, and heart [6, 7]. Compared with traditional processes, this technology provides unparalleled precision and high reproducibility, enabling much faster and precise material preparation, shortening time, and reducing manufacturing costs [8–11]. Thus, 3D printing technology is predicted to lead to the next industrial revolution [12]. Based on the concept of 3D printing, Tibbits from the Massachusetts Institute of Technology first introduced 4-dimensional printing (4D printing) technology at the 2013 Technology, Entertainment Design (TED) conference. The printed rope on display can be automatically folded into a three-dimensional structure with the word Massachusetts Institute of Technology (MIT) when placed in the water, which has since opened the door to the study of 4D printing [13]. 4D printing refers to the fact that the shapes or functionalities of the 3D printed object can change spontaneously and programmatically under a specific external stimulus. This kind of change can be pre-designed, “programmable”, and the type of nature of the change, as well as the manner and extent of the change, can be carried out in the pre-designed manner [14]. 4D printing not only makes the preparation of complex structures possible, but also makes the prepared structures dynamic and intelligent, which is of great significance to shorten the process of bone repair and accelerate bone healing. With the boom in 3D printing and 4D printing, the concepts of 5-dimensional printing (5D printing) and 6-dimensional printing (6D printing) have been proposed in recent years to refine additive manufacturing-technologies and have a wide range of applications. 5D printing uses a five-axis printing technology that not only works on the X, Y, and Z axes, but also print on the other two axes to produce multi-dimensional objects. Unlike 3D-printed objects, 5D-printed objects have stronger mechanical properties and also saving a great deal of raw material [15]. The concept of 6D printing, which is also being introduced for the first time in 2021, can be seen as a combination of 4D and 5D printing. In other words, 6D printed objects have strong mechanical properties as well as being able to respond to external stimuli, a new type of intelligent material [16].

When treating tissue defects such as large bone defects–defects that of 2 cm or more in length or defects larger than 2–2.5 times the diameter of the long bone, caused by high-energy trauma, severe infection, bone tumor removal, and so on, failure rates of bone repair may reach up to 50%, which places a heavy burden on families and society [17, 18]. Failure of bone healing will eventually inhibit the blood supply to the tissue, leading to bone tissue ischemia, osteonecrosis, and bone nonunion, thus making stent implantation inevitable. In recent years, a wide variety of tissue-engineered scaffolds have been designed to repair bone defects. The most commonly used bone graft materials include autologous bones, allogeneic bones, metals, and synthetic materials. However, the application of conventional bone repair materials is limited due to insufficient donors, single structure, and lack of functionality. In addition, bone is a highly vascularized tissue, and the establishment of a vascular network is vital for bone development and repair after bone injury, making the formation and maturation of blood vessels the critical factor for bone regeneration [19–22]. 3D printing technology is an efficient and straightforward technology with unique advantages in the design and regulation of personalization, precision, mechanical strength, and porosity, as well as the complex spatial structure of scaffolds, which can better meet the conditions of vascularized osteogenesis [23–26]. As a result, 3D printing technology is extensively used in bone and other tissue engineering [27]. Compared with the constructs manufactured by 3D printing, the constructs manufactured by 4D printing not only have the three-dimensional structure, but the constructs can also exhibit different shapes at different points in time, which means that 4D printing technology can not only manufacture complex and diverse three-dimensional constructs, but the 4D constructs are no longer static and inanimate, but intelligent and dynamic [28]. The printed objects can change their shapes or functionalities when an external stimulus is imposed or when cell fusion or post-printing self-assembly occurs and adapt to the native microenvironment of the bone defect area, providing a new strategy for bone tissue engineering. In addition to good osteogenic properties, timely and adequate vascularization is also essential for successful bone repair scaffolds. On top of 3D and 4D printing, the introduction of 5D and 6D printing has opened up a new era of intelligent, high-strength vascularized bone repair materials. Therefore, this review focuses on vascularized additive manufacturing-printed bone repair biomaterials.

**Scheme 1:**
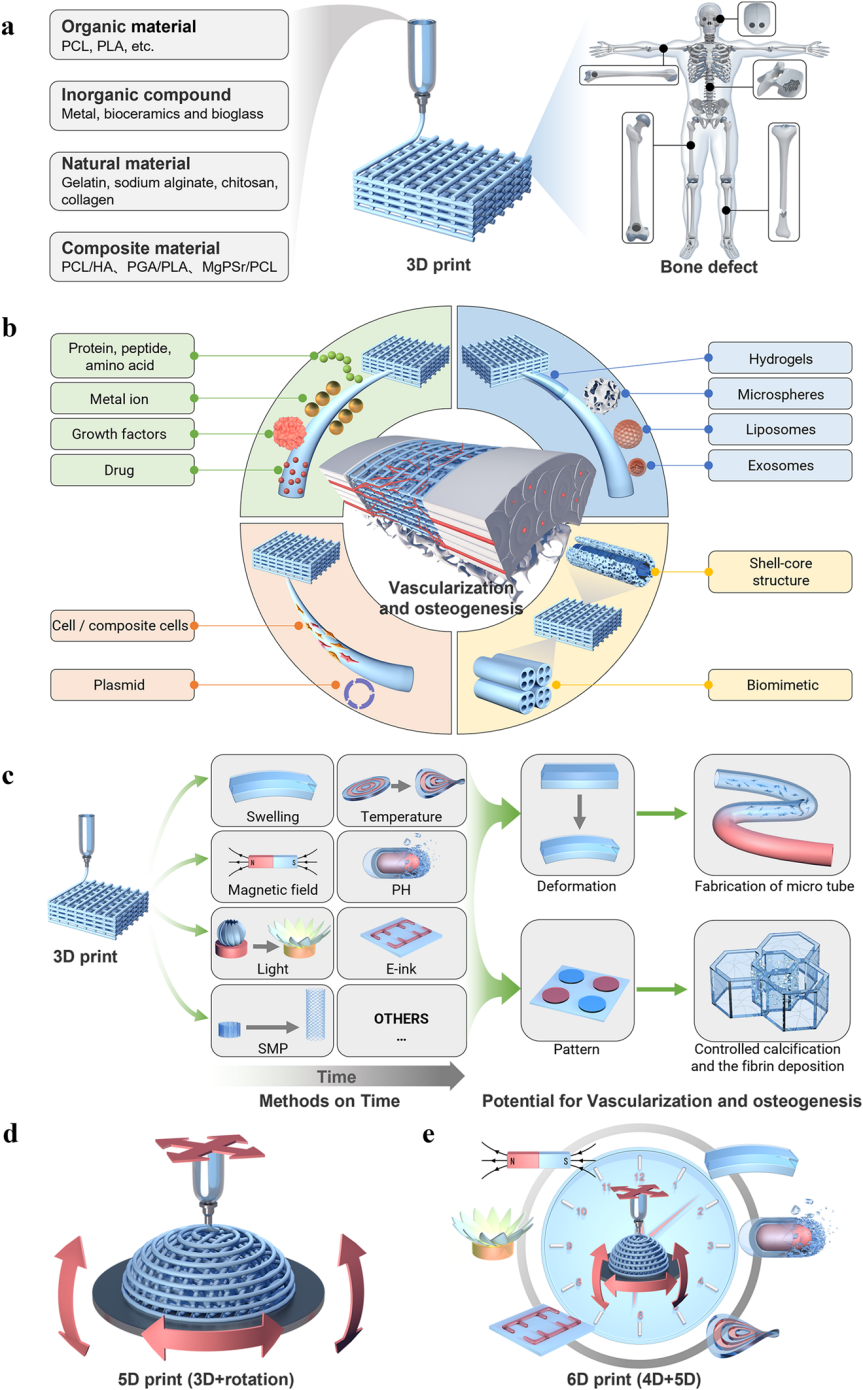
Schematic representation of materials and approaches for intelligent vascularized additive manufacturing-scaffolds. **A** Schematic diagram of the application of additive manufacturing in bone tissue. Three main parts are described: the raw material for additive manufacturing, the printing process and the application scenario. (From left to right) **B** Four representative modifications of 3D vascularized scaffolds: functional vascularized 3D printed scaffolds, vascularized 3D printed scaffolds loaded with specific carriers, bionic vascularized 3D printed scaffolds cell-based and vascularized 3D printed scaffolds. (From the top left, clockwise) **C** The response process of a 4D printed vascularized scaffold. From left to right: the printing process, the processes of dissolution, light, magnetism and temperature over time, the potential for applications in vascularized osteogenesis. **D** and **E** The printing process for 4D and 5D printing

So far, there have been studies on vascularization, 3D printing, 4D printing, 5D printing [29], 6D printing [30], and biomaterials regarding bone regeneration. However, the research on additive manufacturing-vascularization in bone tissue repair and reconstruction is summarized based on the unique entry point of the importance of vascularization in bone repair. Firstly, the development and significance of vascularity in bone tissue is described in four ways: neovascularization and development, specific vascular networks in tissues, vascularization and tissue repair, and vascularization and tissue engineering. Next, the research progress and approaches of vascularized 3D-printed bone repair materials are summarized and presented in four categories: functional vascularized 3D-printed scaffolds, cell-based vascularized 3D-printed scaffolds, vascularized 3D-printed scaffolds loaded with specific carriers, and bionic vascularized 3D-printed scaffolds (Table 1). Additionally, by listing the application examples of 4D printing in vascularized bone tissue engineering and related fields, analyzing the advantages of 4D printing and summarized and prospected the application of 4D printing in bone tissue engineering in the future. Moreover, the vascularized 4D printed scaffolds in the bone were reviewed and the fundamental mechanisms responsible for the functionalities were discussed in detail. Besides, the excellent advantages of 5D/6D printing scaffolds in creating multi-dimensional tissues especially artificial tissues like bones with curved surfaces and much stronger mechanical properties and their challenges in industrialization are discussed. Finally, the related application of vascularized additive manufacturing-scaffolds briefly summarized for future explorations and it drew a conclusion and prospect on the research of vascularized additive manufacturing-bone repair materials (Scheme 1).

**Table 1:** The research progress and approaches of vascularized 3D-printed bone repair materials

Modification	Property	Therapy model	References
**Functionalized 3D printed scaffolds**			
Tantalum scaffold/poly dopamine/magnesium	Sustained ion release enhanced vascularized bone formation	Rat femur condyles bone defect	[96]
PCL/HA scaffold/Sr^2+^/Fe^3+^	Synergetic release of Sr2 + /Fe3 + achieved bone regeneration with immunomodulation, angiogenesis, and osteogenesis	Rat cranial defect	[155]
TCP scaffold/SiO_2_ and ZnO dopants	Addition of SiO_2_ and ZnO to the scaffolds facilitated cellular attachment and proliferation	In vitro cell experiments	[156]
PCL scaffold/surface aminolysis /DFO	Controlled release of DFO achieved the rapid 3D vascularization and bone regeneration	Rat femur defect	[103]
MBG/PHBHHx scaffold/DMOG	Sustained DMOG release enhances the angiogenesis and osteogenesis	Rat bone defect	[157]
β-TCP scaffold/RGD-phage	Stable REG production induced the regeneration of vascularized bone	Rat radius defect	[160]
Mesoporous calcium silicate scaffold/SVVYGLR	The stable and sustained release of SVVYGLR promoted more tubular vessel formation and homogeneous new bone regeneration	Rabbit radial defect	[161]
Hydroxyapatite/calcium sulfate scaffold/VEGF	The stable release of VEGF improved bone regeneration and angiogenesis	Rabbit femur defect	[163]
PLA scaffold/gelatin and Polylysine/BMP-2/VEGF	Sequential release of BMP-2/VEGF in spatiotemporal successfully induced angiogenesis and osteogenesis	In vitro cell experiments	[164]
PCL scaffold/BMP-2/VEGF	Spatially and temporally delivered BMP-2 and VEGF sequentially promoted angiogenesis and bone regeneration	Subcutaneous model of mice	[165]
**Special carrier-loaded 3D printed scaffolds**			
Ti_6_Al_4_V scaffold/poloxamer 407 hydrogel/simvastatin	Sustained release of simvastatin promotes osteogenesis and angiogenesis	Rabbit tibial defect	[174]
Gel/alginate/β-TCP scaffold PLGA microspheres/VEGF	Sustained release of VEGF promotes osteogenesis and angiogenesis	In vitro cell experiments	[178]
β-TCP scaffold/Gel microspheres /Liposome DFO	Controlled release of DFO promotes osteogenesis and angiogenesis	Rat femoral defect	[104]
PCL scaffold/exosomes/VEGF	Delivery and protection of VEGF promoted osteogenesis and angiogenesis	Rat radial defect	[182]
**Cell-modified 3D printed scaffolds**			
PLA scaffold/EPCs/hBMSCs	Improved cell survival, oxygen diffusion, and nutrients. Promoted Osteogenesis and angiogenesis	In vitro cell experiments	[187]
PCL/HA scaffold/hydrogel ADMSC/HUVECs	Accelerated the establishment of vascular network	Subcutaneous model of mice	[189]
PCL/HA scaffold/SVFCs hydrogel	Short-term hypoxia promoted vascularization	Subcutaneous model of mice	[191]
PDACS/PCL/WJMSCs/HUVECs hydrogel scaffold	Promoted the formation of the vascular network and enhanced osteogenesis	In vitro cell experiments	[192]
Hyaluronic acid (HAMA)/alginate/HUVECs microvessels	Injection and suturing, be introduced into large bone repair implants for pre-vascularization and osteogenesis promotion	Subcutaneously in a murine model	[190]
PCL/TCP/hFASCs hydrogel scaffold	Promoted osteogenesis and vasculogenesis	Rat cranial defect	[193]
HUVECs/ASCs hydrogel scaffold	Promoted osteogenesis and vasculogenesis	Mice muscle implantation	[194]
BMP-hBMSCs/GelMA scaffold	Promoted osteogenesis and vasculogenesis	Mice muscle implantation	[195]
Alginate/HA/plasmid MSCs/PCL scaffold	BMP-transfected cells promoted osteogenesis and angiogenesis	Subcutaneous model of mice	[196]
MSCs/EVs/PLA scaffold	Increased the expression of osteogenic and angiogenic markers	Rat cranial defect	[197]
**Bionic 3D printed scaffolds**			
β-TCP scaffold /MSCs/ECFCs Hydrogel	Realized central vascularization and Osteogenesis	Rabbit femoral defect	[210]
OCP/GelMA hydrogel/HUVECs scaffold	Simulated bone structure; accelerated osteogenesis and angiogenesis	In vitro cell experiments	[211]
CDHA/axial vascular pedicle scaffold	Simulated bone structure achieved osteogenesis and angiogenesis	Sheep large bone defect	[212]
PLGA/β-TCP/CMs AV bundle scaffold	Combined of an AV bundle and rhBMP-2	Rabbit intramuscular pocket	[213]
Bioceramics/autologous total bone marrow/femoral vein scaffold	Illustrated the capacity of an intrinsic vascularization by a single vein to support ectopic bone formation	Subcutaneous model of mouse, sheep, rat, rabbit	[214]
AKT hollow-channel scaffold	Multi-channel structure achieved osteogenesis and angiogenesis	Rabbit cranial defect; Rat muscle implantation	[215]
BRT-H scaffold	Hollow-pipe structure with bioactive ions accelerated osteogenesis and vascularization	Rabbit radius segmental defect	[216]
AKT/bio-ceramic/bioactive glass scaffold	Haversian bone–mimicking scaffold promoted osteogenesis and angiogenesis	Rabbit femoral defect	[217]

## Vascularization in Tissue Repair and Reconstruction

Herein, the importance of vascularization for tissues, using the bone tissue as a starting example, is firstly discussed from four aspects: neovascularization and development, specific vascular networks in tissues, vascularization and tissue repair, and vascularization and tissue engineering.

### Regeneration and Development of Blood Vessels in Tissues

**Fig. 1:**
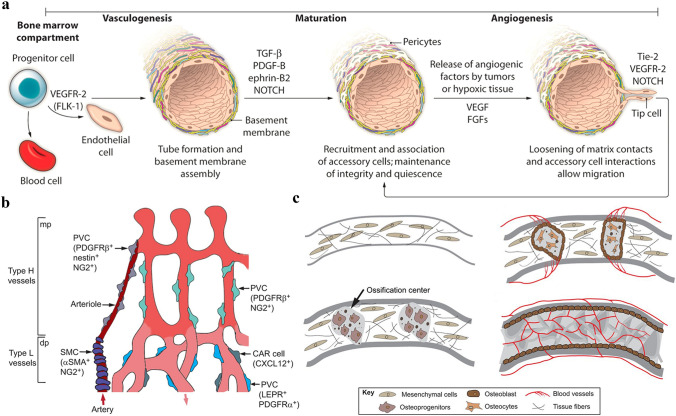
Blood vessel formation and function in the bone tissue. **a** Vasculogenesis and angiogenesis. Two distinct mechanisms of blood vessel formation. Vasculogenesis gives rise to the primitive vascular plexus during embryogenesis. Stimulated by tumors and hypoxic conditions, angiogenesis remodels and expands the vascular network. Reproduced with permission [33]. Copyright 2012, American Association for the Advancement of Science. **b** Perivascular cells associated with blood vessels in the bone. Larger arteries are covered by smooth muscle cells that are connected to the H capillaries while mesenchymal cells are found on sinusoidal type L-vessels. **c** Illustration of intramembranous angiogenesis. Mesenchymal cells condense to form sponge-like structures and differentiate into osteoprogenitors and osteoblasts. Matrix proteins and pro-angiogenic factors generated by the ossification centers then attract blood vessels. **b** and **c** are reproduced with permission [48]. Copyright 2016, The Company of Biologists Ltd

Blood vessels are an important part of the circulatory system in the human body, which deliver nutrients to various organs and tissues, transport metabolic wastes, and establish a connection between tissues [31, 32]. The formation of blood vessels mainly includes angiogenesis and neovascularization [33]. Vasculogenesis is mainly the differentiation and development of endothelial progenitor cells and the formation of a primitive vascular network. In comparison, angiogenesis is the formation of new blood vessels on top of the already existing ones. As for the human body, when the initial vascular network forms into more complex vascular networks, vasculogenesis is followed by angiogenesis thus making it crucial for the repair process of various damaged tissue, including bone defects. Three main types of new blood vessel growth exist: sprouting, non-sprouting, and encapsulated neovascularization [34–38]. Neovascularization is a complex multi-step process, regulated directly or indirectly by many growth factors and cytokines. The basic steps are as follows: first, the matrix enzymes degrade the vascular stromal membrane, and various cytokines stimulate endothelial cells to form new capillary sprouts. Then, the sprouts interconnect with each other to form a new ring structure, which initiates blood flow. The stromal membrane is also remodeled by the interaction of endothelial cells and pericytes, thus promoting maturation and integrity of neovascularization [39, 40]. The vasculogenesis and angiogenesis process for blood vessel formation and maturation is shown in Fig. 1a.

Neovascularization and the development of blood vessels are closely related to the growth and development of the human body and the repair of damaged tissue. The field of vascular biology influences almost all living systems. Bone tissue is a highly vascularized and calcified system in the body, which provides support and protection to other organs and plays an important role in the locomotor system. There is a lifelong dynamic balance between bone resorption and bone remodeling [41–43]. It has been reported that approximately 25% of bone trabeculae and 3% of cortical bone are removed and replaced each year, and it is this constant renewal that also effectively enhances the strength and toughness of the bone and greatly reduces the risk of fracture. This dynamic balance of bone is achieved by the development of a strong vascular network [44–46]. The blood circulation and regeneration of blood vessels are closely related to the normal development, growth, remodeling, and repair of bone. The vascular network in the bone not only serves as a “highway” for transporting nutrients, oxygen, and hormones but also as a “telephone line” for the transmission of signals between bone units and between bone and surrounding tissues.

### Specific Vascular Network in Tissues

The vascular network in bone tissue has a distinct hierarchical and structural specificity [47]. Kusumbe et al. used immunofluorescence labeling to classify this specific vascular network into H- and L-type vessels in bone tissue. H-type vessels are mainly distributed in the metaphysis and endosteum, while L-type vessels are distributed in the medullary cavity of the diaphysis [37, 48]. H-type vascular endothelial cells express significantly more CD31 and endomucin than L-type vascular endothelial cells and are strongly associated with PDGFRß perivascular cells and osteoprogenitor cells that express osterix. The two types of vessels crosslink with each other to form a bone tissue-specific vascular network. More interestingly, when the nutrient artery crosses the bone cortex to reach the medullary cavity, it first flows into the H-type vascular network within the metaphysis and periosteum, then the L-type vascular network, and finally into the central vein near the growth plate [48–50]. As a result, different microenvironments can be detected in different parts of the postnatal long bones, relative hypoxia within the diaphysis, but better oxygenation in the metaphysis. This also explains why bone tissue in the metaphysis is more active than it in the diaphysis. Also, the number of H-vessels decreased with age while the L-vessels did not [35, 48]. The same phenomenon was found in aged and ovariectomized osteoporotic rats, suggesting that estrogen may induce angiogenesis and that the decrease in specific H-type vessels is closely associated with both osteoporosis and bone formation (Fig. 1b).

### Vascularization and Tissue Repair

It is well known that bone defects are mainly repaired through both intramembranous osteogenesis and endochondral osteogenesis. Intramembranous osteogenesis is commonly found in flat bones and irregular bones, while endochondral osteogenesis is common in long bones of the extremities [51–53]. It is emphasized that in either of the osteogenic approaches, osteogenesis and vascularization are closely related in time and space, and vascularization is a prerequisite for osteogenesis [54, 55]. For example, chondrocytes and osteoblasts can release the vascular endothelial growth factor (VEGF), and in turn, endothelial cells can provide signals to osteoblasts. This signaling pathway has a profound effect on bone repair and development [52, 56, 57]. When a fracture or bone defect occurs, the local oxygen supply decreases rapidly, resulting in a hypoxic environment due to the disruption of blood vessels. At the same time, a large number of blood cells form clots for hemostasis at the site of injury [31, 58]. Subsequently, inflammatory cells, fibroblasts, mesenchymal stem cells, and endothelial cells accumulate at the bone defect site [59, 60]. Owing to the early hypoxic environment as well as growth factors and cytokines, non-infectious inflammation and vascularization immediately initiated which are vitally important for fracture healing [61, 62]. The release of growth factors promotes the rapid migration of endothelial cells to the hypoxic area to guide vascular invasion, followed by the proliferation of stalk cells to form an effective vascular network [58, 63, 64]. Moreover, endothelial cells can effectively mediate the migration of osteoblasts and osteoclasts to the site of bone defect, accelerating the process of bone regeneration [65]. Collectively, the repair and homeostasis of the skeletal system are guaranteed by a dynamic balance of osteolysis-osteogenesis accompanied by vascularization, together with the interaction of multiple signaling molecules (VEGF, PDGF, BMP-2, FGF, etc.) and the joint participation of multi-system cells (Fig. 1c).

### Vascularization and Tissue Engineering

Tissue repair is a regeneration process in which most tissue injuries can be restored to pre-injury levels through the recruitment and differentiation of stem cells from the (bone) tissue and vascular tissue, but 10% of the defects remain nonunion [66, 67]. Vascularization plays a crucial role in tissue repair and homeostasis, as (bone) tissues beyond 200 μm from blood vessels in thickness cannot survive properly due to the lack of nutrients, including oxygen and glucose, and the accumulation of metabolic “waste”, such as carbon dioxide and urea [68–70]. When a tissue defect occurs, the vascular networks usually break down, characterized by the reduction of the growth and the reconstruction of the native vascular networks, especially in the central area of the tissue defect, resulting in the insufficient and delayed supply of nutrients [71–74]. Therefore, the rate, range, and density of angiogenesis determine the efficiency of tissue regeneration and tissue defect repair. However, implanted tissue tissue-engineered grafts also suffer from an inadequate exchange of nutrients, resulting in slower cell growth, uneven internal and external osteogenic and vasculogenic induction, even cellular ischemia and necrosis, thus limiting the clinical transformation of tissue engineering materials. Vascular insufficiency is an obstacle to the survival of tissue engineering grafts. Therefore, the main challenge of tissue regeneration is rapid and adequate vascularization, which is a prerequisite for cell survival and implant integration [75–77].

### Vascularization and the Biochemical Microenvironment of Bone Tissue Cells

In the application of vascularized bone tissue engineering, the implantation of vascularized additive manufacturing-scaffolds is used to accelerate the osteogenic process by rapidly building a mature vascular network to facilitate material exchange at the graft site and further stabilize the biochemical microenvironment of bone tissue cells [78, 79]. However, a prerequisite for all this is that the vascularized scaffold must have the mechanical properties required for bone tissue engineering. It is well known that, on a macroscopic scale, typical long bones include cortical bone (dense bone), trabecular bone (cancellous bone or cancellous bone), etc. Cortical bone has a high compressive modulus of elasticity (E ≈ 7.0–30 GPa), high resistance to axial, bending and torsional loads and high compressive strength (Sc ≈ 100–230 MPa) [80, 81]. In contrast, bone trabeculae have a strength of E ≈ 0.1–5 GPa and Sc ≈ 2–12 Mpa [82].This therefore requires the researcher to personalize the vascularized additive manufacturing-scaffold for different tissue sites. In addition, a thorough understanding of the cellular and biochemical composition of bone tissue is required in order to better exploit the role of vascularized additive manufacturing-scaffolds in bone tissue engineering. Bone is a heterogeneous composite material consisting of minerals, collagen (type I) and water. In addition, small amounts of other organic substances such as polysaccharides, proteins, proteoglycans, salivary proteins and lipids are present in this dynamic/vascularized tissue, which give it its tensile, compressive, flexural and elastic properties [83]. Interestingly, bone tissue also includes many bioactive molecules such as alkaline phosphatase, bone marrow matrix protein-2, collagen, elastin, vascular endothelial growth factor (VEGF) and osteocalcin. These active molecules play an important role in the regulation of bone resorption, bone formation, bone repair, bone metabolism and bone growth [84, 85]. Hydroxyapatite (HAp) is the main component of bone minerals and is responsible for providing proper structural support [86]. In addition, the bone has a cellular phase consisting of four main cell types: osteoblasts (bone tissue formation), osteoclasts (bone tissue resorption), osteocytes (bone tissue maintenance) and bone lining cells, which work closely together on the vascular “highway” to carry out a variety of complex life activities through the efficient transmission of signals by active molecules. These cells work closely together to perform a variety of complex life activities through the efficient signalling of active molecules along the vascular ‘highway’. A well-established vascular network is therefore essential [87]. More importantly, bone defects cannot be repaired without osteoblasts, which need to be maintained in a favorable biochemical microenvironment in order to grow and function properly, of which vascularization is an important part of the process. It is the blood vessels that supply oxygen and nutrients and the biochemical microenvironment they provide to bone tissue cells that is essential for their normal growth and development. The growth and development of bone tissue cells is a complex process involving many cellular and molecular interactions [88]. Vascularization is closely related to apoptosis, vascular evolution and bone regeneration.Vascularization occurs through the involvement of a variety of molecular regulators and signal transduction molecules, of which Vascular Endothelial Growth Factor (VEGF) is one of the most critical promoters, a cytokine produced by many tumor cells and other tissue cells that promotes not only intracellular neovascularization but also promote the growth and differentiation of existing blood vessels [89, 90]. How to balance the physiological processes of osteogenesis and vascularization in applications in bone tissue engineering is therefore a challenge for the future development of smart additive manufacturing-scaffolds.

## Vascularization and Graft Materials

Tissue grafting is the traditional approach for tissue defect treatment. Using the bone tissue as an example, the materials commonly used as bone grafts can be classified into four categories: autologous bone grafts, allogeneic bone grafts, metals, and synthetic materials [91–93]. Just as its name implies, the autologous bone graft usually derives from the patient himself to fill the defect area [94]. It is considered the "gold standard" for bone grafting due to its good histocompatibility and non-immunogenicity but consequently causing secondary trauma. Autografts also exhibited limitations in their clinical application due to the high cost, easy deformities, and surgical risks, such as inflammation, infection, bleeding, and pain [95]. Allogeneic bone grafting is another popular option in which the bone tissue graft is derived from a donor, usually from a cadaver. Cheaper though, allografts exhibit much lower osteogenetic capability due to the decreased cellular components after a rigorous sterilization and disinfection process. Besides, being histocompatible to some extent, they still are immunogenic and infectious. Metallic bone graft materials, such as titanium and zinc alloys, are also widely used in clinical practice [96, 97]. There are three significant advantages of metallic materials: first of all, metallic materials can provide required mechanical support by virtue of their inherent advantages. What’s more, as the materials undergo strict sterilization, the risk of infection and immunogenicity is reduced. Finally, materials such as zinc are preferred because of their intrinsic osteoinduction ability. Nonetheless, the life cycle of the implant is often challenged and requires revision surgery, especially in younger patients. In addition, the non-degradability, high stiffness, and poor osseointegration place restrictions on the application of metallic materials. With consideration of the above problems, synthetic materials such as polymers or bioceramics have emerged [98]. The advantages of synthetic materials lie in three main aspects. First, similar to metallic materials, synthetic materials can reduce the risk of infection, disease transmission, and immunogenicity. Second, polymers such as polycaprolactone (PCL) have been widely used because of their superior properties such as better biocompatibility, more modification sites, and sufficient mechanical support. Third, bioceramic such as tricalcium phosphate, whose composition is similar to natural bone, has the advantages of osteoinduction and excellent mechanical properties [99].

Among the four mentioned bone grafting materials, autologous bone grafts are the best in terms of vascularization and osseointegration ability [100]. This is because the skeletal system itself is a large vascular network, and autologous bone grafting is equivalent to transplanting the patient's vascular network to the bone defect site. On the other hand, allogeneic bone grafts can merely provide structural support ignoring the vascularization [101]. As the materials such as metallic materials, polymers, and bioceramics present relatively week performance in vascularization, it’s of great necessity for subsequent modification and assembly to meet this demand. All of the above materials can accelerate the repair of bone defects to varying extents, however, the ideal bone repairing scaffold should be characterized as follows: osteoinductive and osteoconductive properties, vascularization, biocompatibility, personalization, long shelf-life, and low cost. In seeking to address these challenges, tissue engineering has made tremendous progress in promoting the design and structure of scaffolds over the past few decades, particularly by using 3D printing technology to simulate the biological, mechanical, and chemical properties of the target tissue [102]. Thus, it is helpful to create grafts that are more in line with the function and structure of natural bone tissue, minimizing the gap between tissue engineering materials and native tissue, therefore ensuring the effectiveness of material-based bone regeneration, particularly the vascularization of the 3D printed scaffolds is a promising orientation [103, 104].

## 3D printing Technology and Materials

### 3D Printing Technology

**Fig. 2:**
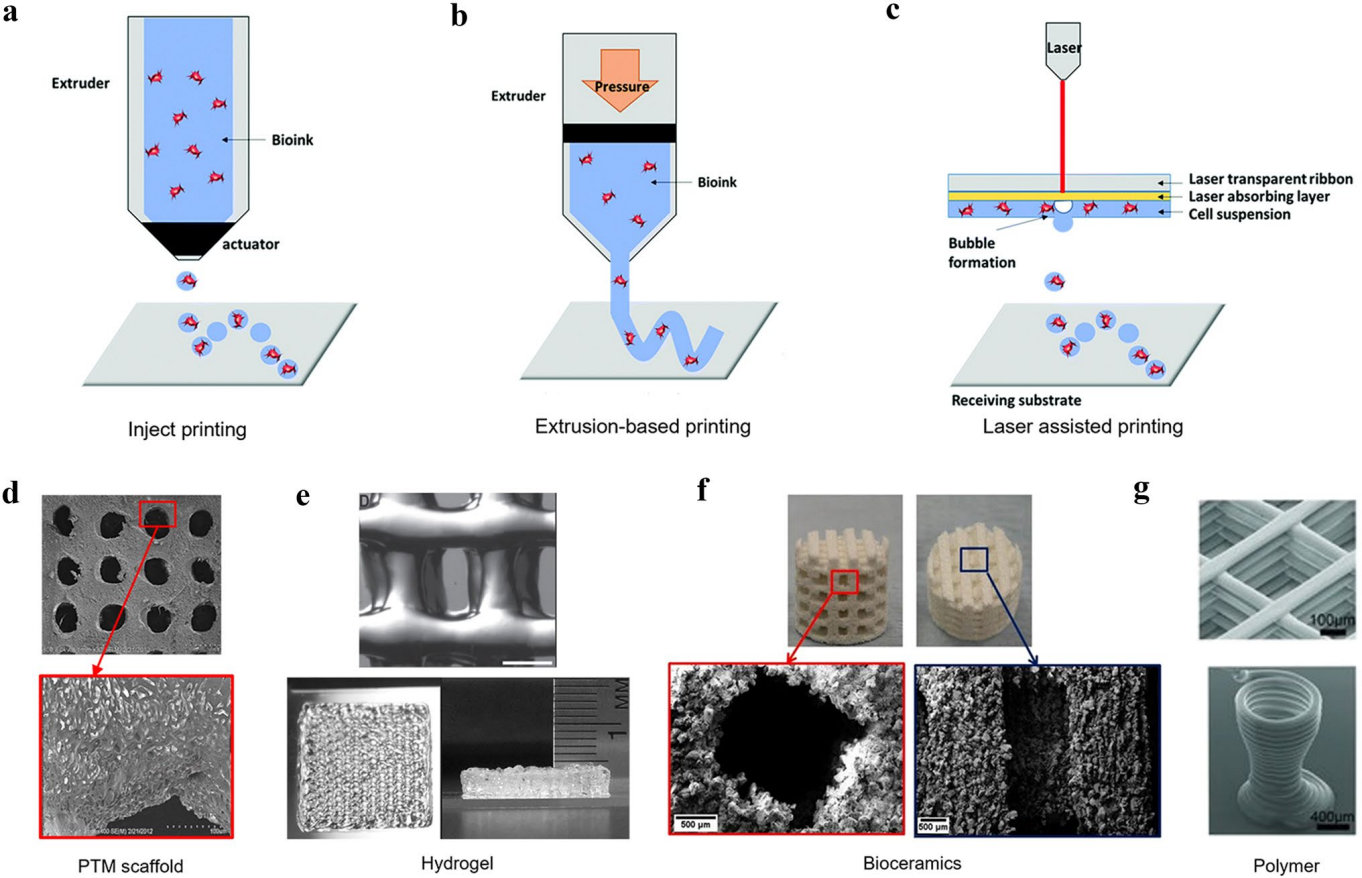
3D printing-compatible techniques. Inject printing (**a**), extrusion-based printing (**b**), and laser-assisted printing (**c**). The biomaterial inks are used in 3D printing. **a**–**c** are reproduced with permission [105]. Copyright 2019, Royal Society of Chemistry. The composite materials (**d**) (Reproduced with permission [123]. Copyright 2019, Elsevier.), natural materials (**e**) (Reproduced with permission [124]. Copyright 2019, Wiley), inorganic materials (**f**) (Reproduced with permission [126]. Copyright 2018, MDPI) and organic materials (**g**) (Reproduced with permission [131]. Copyright 2013, Wiley)

3D printing technology, or additive manufacturing, is the construction of a three-dimensional object from a digital model by stacking layer by layer using adhesive materials, such as powdered metals or plastics [105]. As for the 3D printing process, the first step is to construct a model by computer-aided design (CAD) or other computer software, then “slicing” the established 3D model into layer-by-layer cross-sectional data, generating a file format (usually STL format) that is recognizable by the printer, and finally transmitting this information to the 3D printer [9, 106]. The printer controls the machine to stack these 2D slices based on the description of the ‘sliced’ data, eventually shaping a solid object. 3D printing has many advantages over traditional manufacturing techniques [107, 108]. Using computer-aided design (CAD) technology can simplify the manufacturing process, and computed tomography (CT) and magnetic resonance imaging (MRI) can be applied to collect patient data and create personalized 3D-printed scaffolds [26, 109]. 3D printing technology is simple and efficient in designing and producing scaffolds with unique features, such as personalization, accuracy, mechanical strength, porosity, and complex spatial structures. To date, various ways of 3D printing have been reported, such as droplet-based, extrusion-based, and laser-assisted 3D printing [105] (Fig. 2a–c).

Droplet-based printing has been well developed in the twenty-first century. It is a simple and flexible technique that allows precise droplet distribution on the substrate without contact between the nozzle and the substrate by controlling the droplet. There are three types: inkjet printing, acoustic-droplet-ejection printing, and micro-valve printing. Here, we focus on inkjet printing [110–113]. Inkjet printing is a common 3D printing technology that includes continuous inkjet, drop-on-demand inkjet, and electrohydrodynamic jet printing [114]. It uses a computer-controlled thermal or piezoelectric (acoustic) actuator to generate droplets from the ink in the cartridge and eject the droplets onto the substrate to build 3D structures. The advantages of injecting printing are its fast speed (up to 10,000 drops per second), low cost, and high printing accuracy. However, the major drawback of inkjet printing is that bioink used in printing contains cells, which could cause nozzle clogging and lead to uneven water droplets [115]. Extrusion-based 3D printing is the most widely used method because it is simple, economic, and suitable for mass production [116]. It is capable of delivering a broad range of cells and materials and has a wide range of material selectivity. For extrusion-based 3D printing, hydrogels with cells and polymers (e.g., PCL) are most often adopted. Extrusion-based printers disperse bioink by pneumatic or mechanical systems [117]. These systems physically or chemically solidify 2D patterns, which are stacked to create 3D structures [118]. Such printers can efficiently print highly viscous biomaterials with good cell viability. However, the resolution of extrusion-based printing is very low (~ 200 µm) [119]. Laser-assisted printing, which uses laser beam pulses to print cells, was firstly introduced in the 1940s [120]. During the printing process, the laser pulse creates a high-pressure bubble, which exerts pressure on the bioink layer and forces the bioink to flow to the substrate underneath [121]. The bioink is instantaneously crosslinked as soon as it reaches the substrate. This non-contact printing method separates the inkjet from the bioink, hence there is no contact between the inkjet and bioink, which eliminates the possibility of contamination. In addition, it can maximize the cell viability regardless of high viscosity of the bioink and since there is no nozzle, clogging is avoided [122]. However, the high cost and the limited choice of bioink are the existing disadvantages.

### Materials for 3D Printing

3D printing materials that are often used for bone defect repair can be classified into four categories: inorganic, organic, natural, and composite materials (Fig. 2d–g) [123, 124]. Inorganic materials mainly include metals, bioceramic, and bioglass. As the earliest materials used for the personalized printing of prostheses in the clinic, metallic materials have some unique advantages. Widely used metallic materials include stainless steel, and various alloys like titanium, magnesium, zinc, and cobalt-based alloys [125]. These materials are chemically stable, biocompatible, mechanically strong for bone defect repair. Moreover, some active metal ions such as Mg^2+^, Cu^2+^, and Co^2+^ are not only osteo-inductive but can also promote vascularization in the bone tissue. The second commonest inorganic material is bioceramic, mainly including hydroxyapatite (HAp) and tricalcium phosphate (TCP) [99, 126]. The calcium-phosphorus ratio of hydroxyapatite is 1.667, which is consistent with that in human bone. Being a major component of human bone tissue and teeth, HAp has satisfactory biocompatibility and osseointegration ability. On the other hand, the ratio of calcium to phosphorus of TCP is 1.5, including α-TCP and β-TCP, of which β-TCP is the most widely used. Compared with HAp, β-TCP has better biodegradability and osteogenic properties [123]. On implantation, the calcium-phosphorus ratio in the blood remains normal, even with various ions released from the degradation of β-TCP [127]. However, low mechanical strength and fracture toughness, as well as poor workability are still the big challenges to be overcome. Composed of silicate and phosphate, bioglass shows excellent bone-conduction activity [128]. With 3D printing technology, the shape and size of bioglass can be precisely controlled to manufacture nano-bioglass with a controllable porous structure, excellent mechanical properties, bioactivity, and drug-loading capacity [129]. Nevertheless, bioglass is a fragile material that cannot be used as a primary load-bearing scaffold but is mostly used for bone filling [130].

Organic materials such as polylactic acid (PLA), polycaprolactone (PCL), and polyglycolic acid (PGA), have shown poor mechanical strength but superior flexibility to make it practical for manipulating their properties such as molecular weight and chemical composition, which is beneficial for designing custom-tailored scaffolds [131]. Moreover, the functionality of organic synthetic scaffolds in terms of vascularization and osteoinduction can be further improved by surface modification of the 3D-printed scaffolds [103]. Natural materials—gelatin, sodium alginate, chitosan, collagen, possess good biocompatibility, biodegradability, and low immunogenicity, providing a more favorable microenvironment for cell attachment and proliferation [132]. In bone tissue engineering, natural materials are mostly used as carriers of boink, however, its singular application is limited by the under demand mechanical strength for supporting bone tissues [133]. A composite material is a combination of two or more metals, organic, inorganic, or natural components with a specific ratio. Although each material has its unique advantages, composite materials are before manufacturing versatile and personalized 3D-printed scaffolds [123]. Composite materials such as PCL/HA, PGA/PLA, and MgPSr/PCL have been extensively used in bone tissue engineering [134]. Such combination can impart a certain mechanical strength to the scaffold and also provide maximum functionality to meet the requirements for bone repair.

Bioink is a mixture of cells, biomaterials and bioactive molecules and is widely used in bone tissue engineering. Bioink provides stable 3D structures to promote tissue development and maturation and also mimic tissue microenvironments in situ [25, 135]. 3D printing technology can combine two or more types of bioink, yet more research is needed to demonstrate how bioink can be applied to modulate cellular fate in a better way. An ideal bioink should have the following characteristics [136]. (1) Printability: The bioink must have rheological and phase transition properties that conform to its adapted bioprinting technology to provide the highest possible printing accuracy. (2) Cytocompatibility: Both the composition and the degradation products of bioinks must meet the conditions for living cells to survive and minimize the impact on cell growth and differentiation. In the printing process, the bioink must provide a certain degree of protection for living cells and other biologically active components and protect the cell viability from pressure, shear stress, and various chemical reagents s during the printing process. (3) Versatility: The external intervenes of the bioink, such as the printing method, cross-linking method, and type of bioactive loadings, should be adjustable enough to meet the complex requirements of tissue engineering applications [137]. Bioink is a solution of biomaterial containing living cells and is a key component of bioprinting [138]. It is important that the material present in the biomaterial solution protects the cells from stressors during the printing process [139, 140]. The four main types of bioink materials are hydrogels, microcarriers, cell aggregates and extracellular matrix proteins [141, 142]. Natural polymer components in bioinks include: including gelatin, hyaluronic acid, silk protein and elastin. Synthetic polymers include: amphiphilic block copolymers, PEG, poly (PNIPAAM) and polyphosphonitrile. (1) Polymer hydrogels: Hydrogels are cross-linked hydrated networks of natural or synthetic polymers that are highly biocompatible and biodegradable and do not cause rejection reactions or other adverse effects. In recent years, this type of hydrogel type has been widely used for the repair and growth of vascularized bone tissue [143]. Polymeric hydrogel type bio-ink materials can have their physical and chemical properties modified to meet specific application requirements by adjusting the degree of cross-linking, monomer type and concentration. In addition, polymeric hydrogel-type bioink materials confer the biological signals required for tissue engineering, helping to treat bone defects and promote the growth of vascularized tissue. However, the mechanical strength of hydrogel-type bioinks is inferior and polymeric hydrogel-type bioink materials generally have a lower mechanical strength than other materials such as metals and ceramics, a shortcoming that may limit their use in certain applications (e.g., in areas of bone defects that carry greater weight). In conclusion, polymeric hydrogel type bioink materials have a range of advantages and disadvantages in vascularized bone tissue [144, 145]. Application design requires attention to the modulation of material properties and consideration of biodegradation behavior in order to maximize their therapeutic effect and to achieve their maximum potential in the reconstruction and growth of vascularized bone tissue. (2) Microcarrier-type bioinks allow seeded cells to expand extensively while forming multicellular aggregates and also exert phenotypic control on seeded cells. These effects of microcarriers are mainly achieved by anchoring substrates of dependent cells [146]. The material of the microcarriers can be synthetic polymers, glass or natural polymers such as cellulose, gelatin or collagen. Most importantly, due to its porous spherical form, cells can attach to the surface of the microcarrier and proliferate [147]. In addition, the porous morphology also improves the transfer of gases and nutrients and allows a larger surface area for cell attachment. It is well known that the core of vascularization is to greatly improve the exchange of materials between the graft and the host, to maintain the balance of the local microenvironment of the bone injury and to accelerate the bone repair process [148]. (3) The cellular aggregates type of bioink is a scaffold-free alternative to bioink and consists mainly of tissue spheres, cell granules and tissue chains, where the type I transmembrane protein calmodulin causes intercellular adhesion and cell aggregation, which is important for the efficient establishment of intercellular material exchange and a vascularized network. In addition, the cell aggregate type of bioink material provides a favorable micro-environment that contributes to the survival and proliferation of osteoblasts, thus promoting the repair and regeneration of bone tissue. The bioink material of the living cell type has histological similarities that allow for cellular guidance, allowing new bone tissue to form reliable connections with surrounding cells and tissues, further promoting vascularization and thus improving bone tissue repair and regeneration [7]. However, it is worth noting that the cell aggregation type of bioink requires high cell quality, the low survival rate of the printed live cells and the difficulty in controlling the consistency of the finished product during industrial production due to a combination of cell, printing parameters and environmental issues are all issues that need to be addressed in the future. (4) Extracellular matrix protein is a mixture of active component proteins left over after elution of cells by surfactants and can be obtained from a variety of tissues [149, 150]. Its main function depends on the type of primary tissue, different tissues have different functions and can be obtained from tissues such as skin, adipose tissue, cartilage, bone and heart. The microenvironment formed by extracellular matrix protein types of bioink is important for cell differentiation and function and can be directed to induce differentiation into target tissues. However, it is worth noting that for harder material tissues, such as bone and cartilage, ceramic materials or composite scaffolds are still required to produce mechanically stable scaffolds.

## Functionalized 3D-Printed Scaffolds Promote Vascularization in Tissues

The establishment of a vascular network has an important role in bone growth and bone regeneration [72]. Although the porous structure of 3D-printed scaffolds has positive implications for bone repair, the lack of surface-active groups reduces the ability of scaffolds to vascularize, thus hindering the overall bone repair process. Introducing bioactive groups is an effective way to impart the inert surface with desirable biological properties to address this issue [151–153]. Researchers have been working on modifying active groups onto scaffolds through physical doping and chemical approaches to endow 3D-printed scaffolds with vascularization capability [154]. The reported functionalization methods so far include metals, active drugs, protein or peptides, and growth factors.

### Metal-Modified 3D Printed Scaffolds for Vascularization

**Fig. 3:**
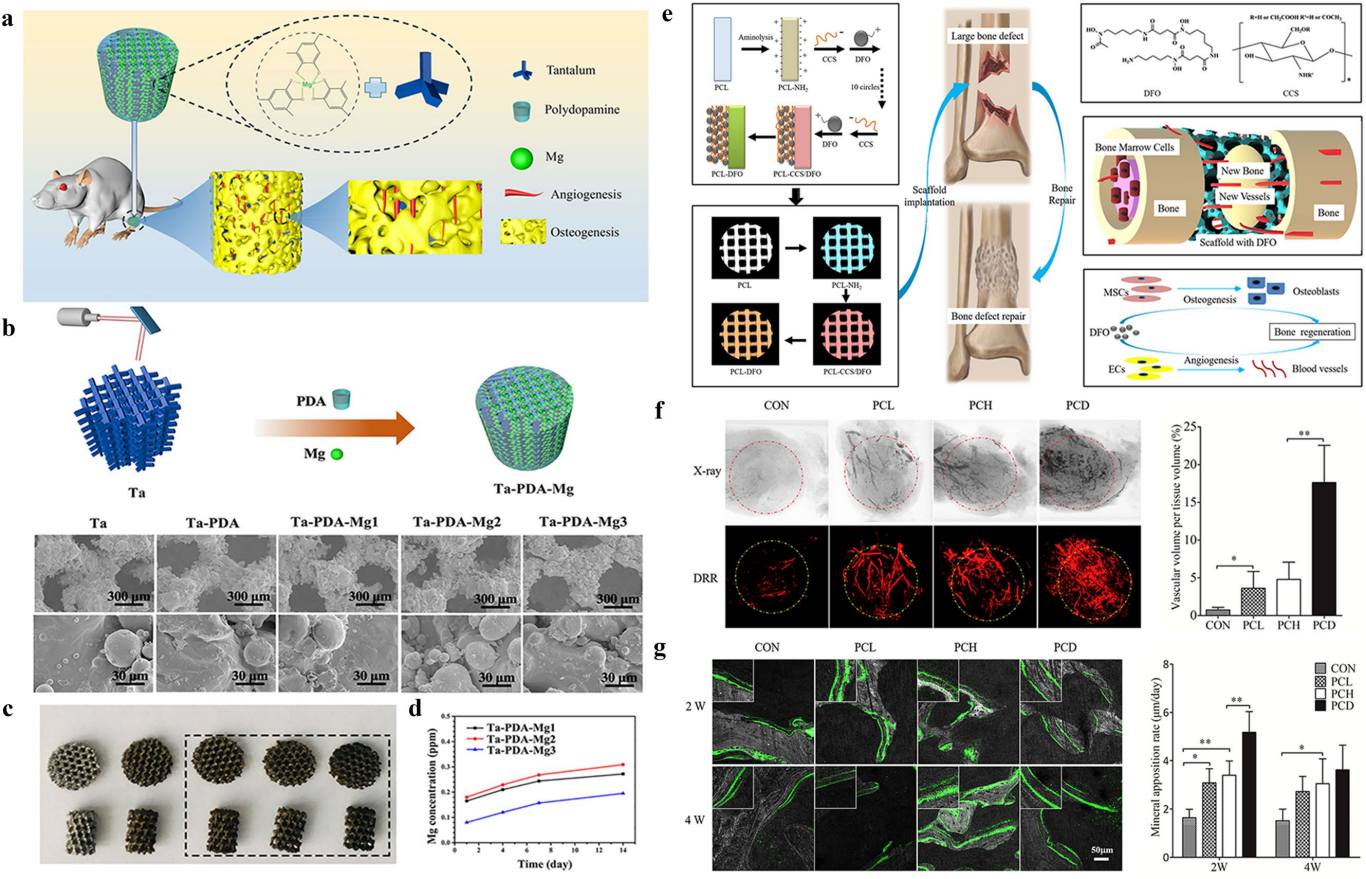
Metal-based and drug modification approaches for vascularized 3D printed networks. **a** Schematic illustration of metal ions modification onto the 3D printed scaffolds. **b** Schematic presentation of the fabrication process and SEM of the samples. **c**, **d** Optical images and cumulative release profile of metal ions. **a**–**d** are reproduced with permission [96]. Copyright 2020, Elsevier. **e** Schematic diagram of DFO on the surface of 3D printed PCL scaffold and its biological function for bone regeneration in bone defect model. **f** Typical X-ray photography and digitally reconstructed radiograph (DRR) after micro-CT scanning of the new vascular formed inside the bone defect site. **g** Dynamic histology of the newly formed bone using double calcein labeling after scaffolds insertion and the summarized data showing the difference of the mineral appositional rate among different groups. **e**–**g** are reproduced with permission [103]. Copyright 2018, Elsevier

The metal-modified 3D-printed scaffolds are becoming increasingly attractive and promising in bone tissue engineering. Among the various approaches, introducing active metals, such as Cu, Zn, Ca, and Mg is an effective way to render inert surfaces with the desirable vascularization properties. Among the metals, magnesium (Mg) is an essential element in bone development good osteogenic and angiogenic abilities. Ma et al. Used the self-polymerization process of dopamine to modify 3D-printed titanium (Ta) scaffolds with magnesium ions and polydopamine to construct the Mg–PDA–Ta scaffolds, which exhibited good biocompatibility and a high ion release rate (Fig. 3a–d). Magnesium ion doping significantly improved the scaffold's in vitro adhesion, angiogenesis, and osteogenic ability. Ta–PDA–Mg significantly promoted vascularized bone formation in a rat femoral condyle bone defect model, thus ensuring more rapid and durable osseointegration of the porous scaffold [96]. Using extrusion-based cryogenic 3D printing and lyophilization, Yang et al. reported novel gradient SrFeHA/PCL scaffolds in another study. As expected, these scaffolds exhibited a 3D interconnected porous structure and rough microsurfaces, as well as a controlled release of bioactive Sr^2+^/Fe^3+^ from the SrFeHA components [155]. With these advantageous properties, the printed scaffolds achieve practical biological applications in vitro and in vivo. They not only directly promote MC3T3 osteogenic differentiation and HUVEC angiogenic function but also manipulate favorable macrophage activation to simultaneously promote osteogenesis/angiogenesis. In addition, the superiority in determining immune response, vascular regeneration, and in situ osteogenesis was further confirmed by the results of in vivo subcutaneous implantation and cranial defect repair. Using 3D printing technique, β-TCP porous scaffolds containing SiO_2_ and ZnO with a pore size of approximately 317 µm were prepared by Fielding et al. The transplantation of the scaffold material to femoral defects in nude mice showed that a large amount of type I collagen and osteocalcin was produced on the scaffold after four weeks. The β-TCP porous scaffold containing SiO_2_ and ZnO significantly promoted the growth of new vasculature and bone formation compared with the single β-TCP porous scaffold [156].

### Surface Drug-modified 3D-PRINTED Scaffolds for Vascularization

Small molecular drugs for angiogenesis and vascularization are widely applied in bone tissue engineering, either by carrier delivery or by modification onto the scaffold directly. Examples of carrier delivery will be elaborated in the next section, while this section will focus on the examples of surface modification. For example, Yan et al. successfully prepared a 3D-printed biodegradable scaffold by modifying the scaffold surface by desferrioxamine (DFO), a small-molecule compound with pro-angiogenic and pro-osteogenic functions, using surface ammonolysis and layer assembly techniques [103]. The release of DFO could be controlled by the modified scaffold, which is necessary for angiogenesis and osteogenesis and matches with bone development and reconstruction. The scaffold significantly promoted the formation of the vascular configuration of human umbilical vein endothelial cells, the production of mineralized matrix, and the expression of osteogenic-related genes during the osteogenic differentiation of bone marrow mesenchymal stem cells (hBMSC), as has been shown in vitro studies. In vivo experiments showed that vascularization and bone regeneration at the defect site in a rat bone defect model could be promoted by the modified scaffold (Fig. 3e–g). Zhu et al. designed a composite scaffold (MPHS) consisting of dimethylallyl glycine (DMOG), mesoporous bioactive glasses, and polymer poly(3-hydroxybutyrate-co-3-hydroxyhexanoate) by physical doping and investigated whether the sustained release of DMOG promotes local angiogenesis and bone healing. The DMOG release patterns of scaffolds loaded with different DMOG doses were evaluated in vitro. Furthermore, they investigated the effects of DMOG delivery on hBMSC adhesion, proliferation, osteogenic differentiation, and angiogenesis-related gene expression. In vivo experiments were performed to evaluate the effect of composite scaffolds on vascularization and osteogenesis. The results showed that DMOG was consistently released from MPHS scaffolds over four weeks and significantly enhanced the angiogenesis and osteogenesis in the defects [157].

### Protein/Peptide-Modified 3D Printed Scaffolds for Vascularization

It has been demonstrated that surface modification by encapsulation of fibronectin, collagen, or laminin can alter the surface morphology of the materials. As a result, the material can modulate the cellular behavior to influence the microstructure and biological function of the tissue it forms. Klein et al. modified the surface of 3D-printed scaffolds with bone sialoprotein (BSP) by physisorption based on the high affinity of CaP biomaterials for proteins [158]. BSP is a non-collagenous protein of the extracellular matrix (ECM) that can bind to hydroxyapatite via its specific binding sites. BSP triggers the mineralization of the ECM and can attract osteoblasts to promote osteoblast adhesion and differentiation via the vitronectin receptor (αvβ3). BSP also promotes endothelial cell migration, attachment, and angiogenesis. Physisorbed BSP has slow-release kinetics in vitro and effectively promotes the osteogenic differentiation of primary human osteoblasts and the formation of endothelial cell (ECs) vascular networks.

Compared with intact proteins, peptides are more capable of precisely control the biological effects they induce and easier to act on substrates. When applied for medical treatment, the advantages of peptides are rapid metabolism, low risk of the immune response, and small molecular weight. Specific peptides can promote the adhesion of endothelial progenitor cells on their surface through specific peptide sequences of adhesion proteins or extracellular matrix molecules, thereby reducing the rolling velocity of ECs when in contact with them, supporting dynamic adhesion and triggering vascularity. For example, αv-containing integrins are highly expressed in activated ECs in the vasculature during trauma healing, which is crucial for vascular regeneration. This integrin regulates ECs migration and apoptotic adhesion by explicitly recognizing the Arg-Gly-Asp (RGD) peptide in the ECM, thereby promoting neovascularization [159]. Based on this, Wang et al. first constructed a biphasic calcium phosphate (BCP) bionic scaffold using hydroxyapatite (HA) and β-tricalcium phosphate (β-TCP) with a mass ratio of 60/40. Subsequently, RGD-phage nanofibers were constructed by a genetic engineering approach. Then, RGD-phage nanofibers (negatively charged) were stably bound to chitosan (positively charged) by electrostatic interactions to be attached to the surface of the 3D-printed bioceramic scaffold. In a rat radial defect model, micro-CT showed that the metric of the bone bulk density almost reached normal control levels, and the internal vascularization in the scaffold was successfully induced [160]. SVVYGLR peptide is an important peptide fragment of osteopontin (OPN), which has the advantage of a small molecular weight. This peptide is expected to stimulate neovascularization and improve internal ischemia of the bone repair material. Unlike RGD, which has a non-specific pro-adhesive effect, SVVYGLR is a specific pro-vascular peptide. Although it could not promote the proliferation of ECs, it significantly enhanced their adhesion and lumen formation activity. Its vascularization effect is comparable to or even more potent than that of VEGF. The OPN-derived SVVYGLR peptide with dual pro-vascularization and pro-osteogenic effects was covalently bound to the surface of mesoporous calcium silicate (MCS) powder material to activate the scaffold material, thereby constructing an SVVYGLR-MCS composite scaffold by 3D printing technology. The SVVYGLR-MCS artificial bone scaffold exhibited good mechanical properties and is suitable for bone defect repair. In vitro, the composite scaffold is beneficial for the osteogenic differentiation of rat BMSCs, adhesion of HUVECs, and lumen formation. In vivo, the composite scaffold has better internal osteogenesis and vascularization than the MCS group in the repair of bone defects [161].

### Growth Factor-Modified 3D-Printed Scaffolds for Vascularization

Many growth factors, such as angiogenic and osteogenic factors are involved in the bone regeneration process. Therefore, integrating various growth factors into 3D-printed scaffolds to build vascular networks is another effective bone tissue engineering strategy. Growth factors are involved in the regulation of different stages of bone regeneration, for example, regulating cellular responses accelerating the formation of new tissues, etc. Physically encapsulating covalently or non-covalently binding growth factors into scaffolds can effectively promote the ex vivo osteogenic and angiogenic capacity of 3D-printed scaffold materials. The widely used growth factors include vascular platelet-derived growth factor (PDGF), endothelial growth factor (VEGF), fibroblast growth factor (FGF), epidermal growth factor (EGF), erythropoietin (EPO), transforming growth factor (TGF), hypoxia-inducible factor (HIF)-1, bone morphogenetic protein-2 (BMP-2), and BMP-7 [162].

**Fig. 4:**
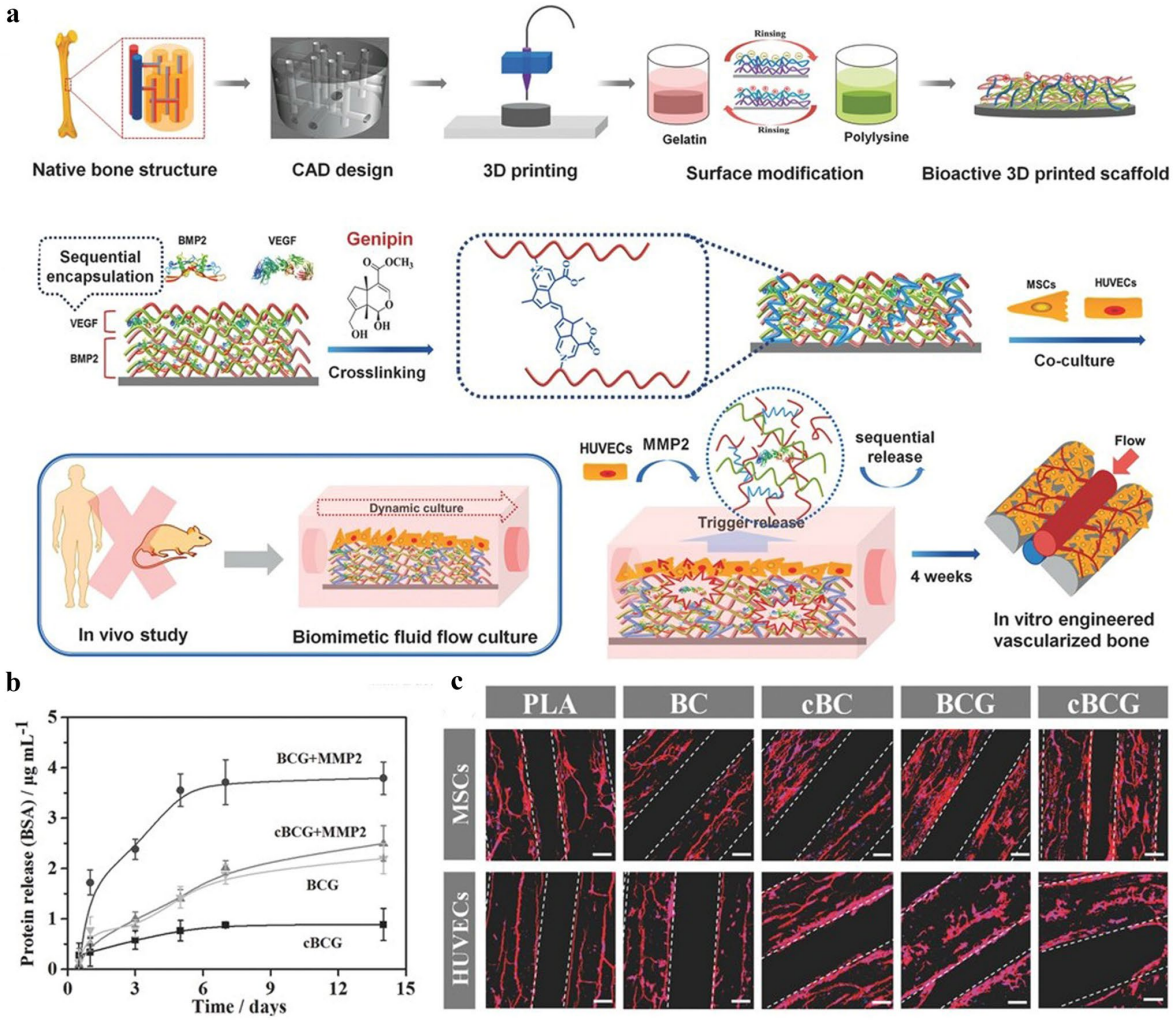
Surface functionalization through polypeptide and growth factors grafting.** a** Schematic representation of the fabrication process of nanocoating modified 3D printed scaffolds and the sequential release of growth factors in the nanocoating film. **b** Protein release profiles of nanocoating within 2 weeks. **c** Fluorescent images of hMSCs and HUVECs on the 3D printed scaffolds. The hMSCs exhibited a well-distributed spread on scaffold surface, while the HUVECs formed an aggregative microvascular networks. ** a**–**c** are reproduced with permission [164]. Copyright 2016, Wiley

Among these growth factors, VEGF is considered to be the most critical signaling molecule for stimulating angiogenesis. It is an important regulator of physiological angiogenesis during embryogenesis. In addition, VEGF promotes intramembranous and endochondral ossification by inducing neovascularization. The hybrid mesoporous 3D-printed scaffold hydroxyapatite/calcium sulfate (HACS) was fabricated by Chen et al. using hydroxyapatite, calcium sulfate, and polycaprolactone. This scaffold was then modified with a VEGF. They recorded the in vitro release profile to show that the HACS scaffold was able to release VEGF in a time-dependent manner. The in vivo experimental results in a rabbit femoral bone defect model revealed that HACS/VEGF has good osteogenic induction [163]. In another related study, a hydroxyapatite porous scaffold was prepared by low-temperature 3D printing and layer-by-layer (LBL) assembly, followed by loading BMP-2 and VEGF onto the 3D-printed scaffold by simple adsorption. Experimental results suggest an excellent osteogenic and angiogenic ability of the BMP-2/VEGF composite scaffold to promote new bone generation (Fig. 4a–c) [164]. By adopting 3D printing technology, Park et al. fabricated a BMP-2 and VEGF-loaded scaffold, with BMP-2 loaded in the peripheral region of the scaffold and VEGF in the central region. This scaffold allowed for sustained release of BMP-2 but the rapid release of VEGF to achieve early vascularization and late osteogenesis for bone defect repair [165].

## Special Carrier-Loaded 3D-Printed Scaffolds for Tissue Vascularization

Osteogenesis is a process closely coupled with vascularization, and recent researches have focused on achieving effective and complete vascularization in 3D-printed scaffolds. Most recent studies have reported that combining active small molecules with 3D-printed scaffolds through suitable carriers to obtain a sustained and stable controlled release profile and endow the scaffolds with vascularization ability [166–168]. Commonly used active small molecules include drugs (DFO, simvastatin) and growth factors (VEGF, BMP-2) [169]. Widely used carriers can be broadly classified into three main categories: hydrogels, microspheres, and nanoparticles (liposomes, exosomes, etc.).

### Hydrogel Carrier-Modified 3D-Printed Scaffold

Hydrogels are formed by one or more monomers that are cross-linked into a net structure by a simple reaction. Their application in the biomedical fields has been extensively developed since 1960 when hydrogels were first used to treat ophthalmic diseases [170, 171]. The highly hydrated nature of hydrogels makes them very biocompatible. Owing to their excellent mechanical properties, hydrogels have attracted wide attention in biomedical fields, such as bone and cartilage repair, wound dressing, wound healing, cell culture, and nerve repair. Hydrogels are also an excellent carrier for drug and small molecule delivery. The structure of hydrogels is porous, and the internal three-dimensional network is interconnected, such that the polymer molecules are easy to be modified. These properties make hydrogels immensely versatile for drug delivery [172]. On the other hand, hydrogels have superior hydrophilicity and injectability and can construct a controlled-release drug delivery system to overcome the disadvantages of traditional drug therapy, which requires high drug doses or multiple doses for treatment [173]. This superiority may improve the therapeutic effectiveness, minimize unnecessary pain for patients, and reduce the risk of toxic side effects. Liu et al. successfully prepared a hydrogel-based 3D-printed composite scaffold by injecting a simvastatin-encapsulated poloxamer 407 thermosensitive hydrogel, which serves as a controlled release system for vascularized drugs, into a 3D-printed porous titanium scaffold. Simvastatin was continuously released from the scaffold for up to 11 days in vitro, which was greatly superior to conventional delivery systems. The effects of the composite material on osseointegration, bone ingrowth and neovascularization were evaluated in vivo using a rabbit tibial defect model. It was revealed that the composite scaffold significantly improved neovascularization, osseointegration, and bone repair due to the sustained release of simvastatin and the osteogenic effect of 3D printing [174].

### Microsphere Carrier-Modified 3D-Printed Scaffold

In recent years, micro-nano drug delivery systems have consistently drawn attention and made rapid progress, especially in pharmaceutical and materials science, where micro-nano carriers of different sizes, structures, and functions have been designed [175]. They mainly include microalgae, microspheres, liposomes, and exosomes. These carriers are small, usually ranging from a few nanometers to a few hundred microns, and are injected into patients by the intravenous or local administration [176]. They not only protect the drug activity from being altered or excreted from the body but also extend the blood circulation time and improve the stability of the drugs, enhance drug accumulation, improve the killing efficiency of the drugs while increasing the drug tolerability, and even enable synergistic treatment with multiple drugs [92, 177]. Fahimipour et al. used a double emulsion solvent evaporation technique to prepare poly(lactic-co-glycolic) acid (PLGA) microspheres to encapsulate VEGF and subsequently doped PLGA microspheres into gelatin/alginate/β-TCP to form a 3D printing ink to print and fabricate custom-tailored bone repair scaffolds. In vitro release experiments demonstrated the sustained release of VEGF from PLGA microspheres within ten days, and the biological concentration was sufficient for vascularization. Apart from that, the human umbilical vein endothelial cells (HUVECs) and osteoblasts that inoculated on the scaffold also exhibited good cell viability, and the ALP activity was greatly increased because of the presence of VEGF. This study once again elucidated the significance of vascularized osteogenesis [178].

### Liposome Carrier-Modified 3D-Printed Scaffold

**Fig. 5:**
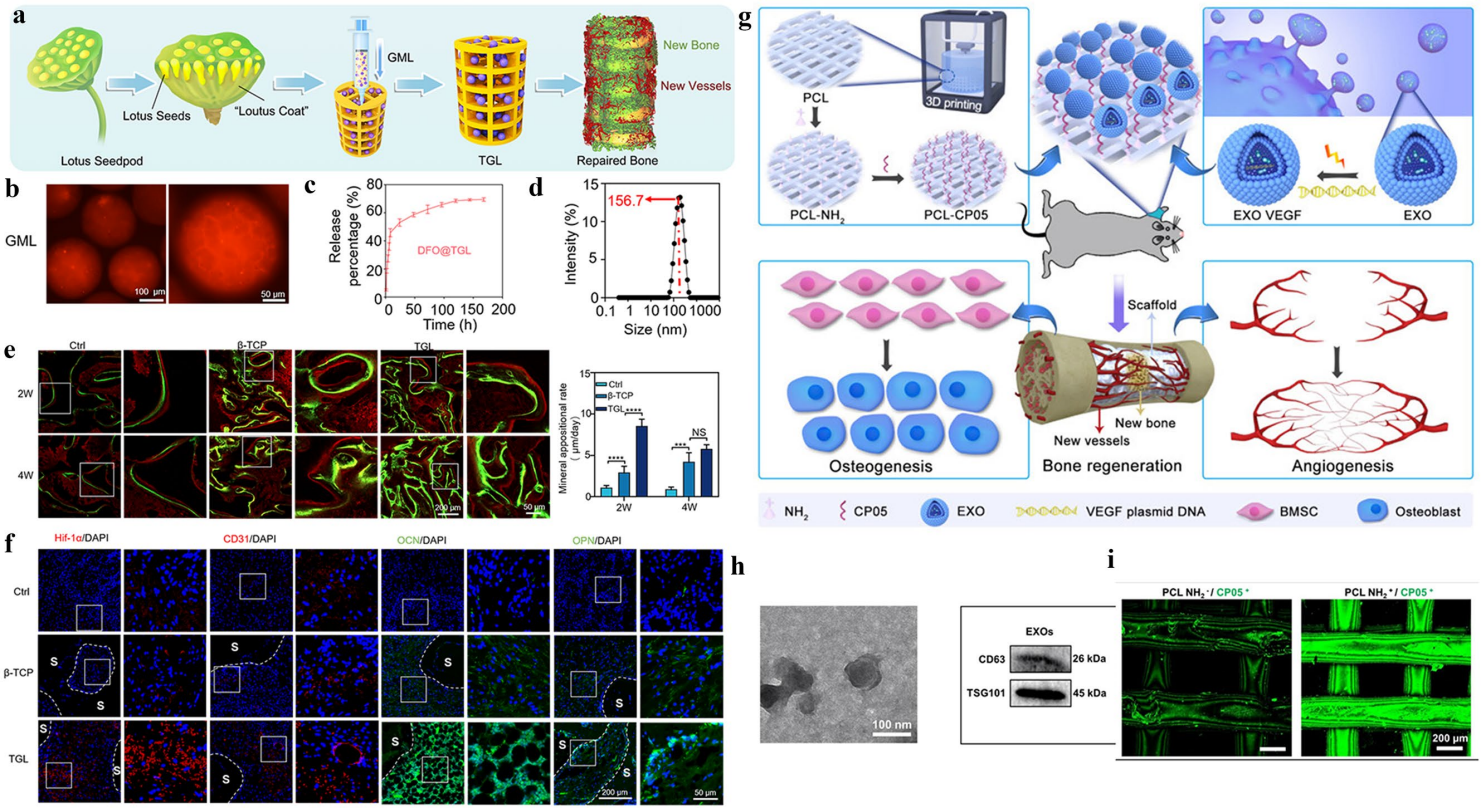
Special carriers loaded 3D printing scaffolds. **a** The schematic representation of designing a lotus seedpod-inspired internal vascularized 3D printed bioceramic scaffold with internal vascularization and osteogenesis functionalities. **b** Fluorescent microscopic images of GelMA Microspheres. **c** The in vitro release profile. **d** Characterization of liposome particles via DLS. **e** The double calcein labeling and the quantitive results. **f** Immunofluorescence staining of the angiogenesis and osteogenesis related genes A-F are reproduced with permission [104]. Copyright 2021, Elsevier. **g** General illustration of engineered exosome enhanced therapies on osteogenesis and angiogenesis. **h** TEM images and protein markers of exosomes. The immunofluorescence image of modified scaffold and the exosomes. **g**–**i** are reproduced with permission [182]. Copyright 2021, Ivyspring International Publisher

Liposomes are self-assembled vesicles of lipid–water mixtures that are similar to cellular transit vesicles in which the lipid components consist of phospholipids and cholesterol [179]. A lipid bilayer is formed under certain interventional conditions (usually ultrasound), which render liposomes the ability to encapsulate both hydrophilic and lipophilic drugs [180]. Therefore, liposomes are considered to be the most classic nano-drug delivery system [181]. Being non-toxic, non-immunogenic, and fully biodegradable drug carriers with the ability to control drug release, liposomes also can reduce side effects. Our previous work was inspired by the special biological structure of “lotus seedpod” and the concept of internal vascularization, a porous bioceramic scaffold was constructed as the “coat” of “lotus seedpod” by 3D printing (Fig. 5a–f). DFO-loaded liposomes were physically combined with microfluidic hydrogel microspheres and became “lotus seeds” by immersion adsorption. Then, the “lotus seeds” were directly injected into the interior of the 3D-printed scaffold to construct an internally vascularized bioceramic 3D-printed scaffold to facilitate the repair of bone defects. It’s observed that DFO s was released in a sustained manner from the composite scaffold in vitro for up to seven days, which is significant for reducing the toxic effects of DFO and continuously inducing vascularized osteogenesis. The composite scaffolds has also shown excellent osteogenic ability in subsequent in vitro and in vivo experiments [104].

### Exosome Carrier-Modified 3D-Printed Scaffold

Exosomes are nanoscale extracellular vesicles composed of proteins, nucleic acids, and lipids, which are widely found in various biological fluids, such as blood, saliva, and urine. Inside the exosomes, a variety of content molecules such as proteins, mRNA, non-coding RNA, DNA, and lipids are present [169]. Exosomes have attracted great interest in the field of regenerative medicine, for they are recognized as an ideal nanocarrier for drugs due to their overall biocompatibility, good tolerability, and low toxicity, as well as their immune escapability [167]. The conventional delivery of small molecular proteins and genes, such as VEGF and BMP-2, in bone tissue engineering, has some limitations, such as uncontrollable release, need for high doses with a short half-life, and low transfection efficiency. Therefore, exosome-loaded small molecular active protein systems are particularly favored. Zha et al. used ATDC5-derived exosomes to encapsulate the VEGF gene and efficiently combined exosomes with 3D-printed scaffolds via the specific exosome-anchoring peptide CP05 (Fig. 5g–i). In vitro experiments showed that exosomes can not only modulate the release of VEGF but also promote the differentiation of bone marrow mesenchymal stem cells toward osteogenesis. In vivo experiments using a rat radial defect model demonstrated that the exosome-mediated engineered bone scaffold could effectively induce massive bone regeneration with vascularization [182].

## Cell-Modified 3D-Printed Scaffolds for Vascularization in Tissues

An ideal bone scaffold material should not only have good biocompatibility, bioactivity, biodegradability, and mechanical support but also needs to satisfy the demands for vascularization and bone growth [183]. Scaffolds and seeded cells are essential for the construction of biologically active tissue engineering scaffolds that can be rapidly vascularized [184]. In bone tissue engineering, the most extensively used cells to induce vascularization for osteogenesis are mesenchymal stem cells (MSCs) and ECs. The primary sources of MSCs are bone marrow, adipose tissue, umbilical cord, and placenta [185]. Here, we focus on BMSCs, whose biological properties are embodied in the osteogenic differentiation ability, regenerative potential, directional migration ability, and the production of the corresponding growth factors and cytokines to promote the repair of bone injury [186]. Endothelial cells are essential cells in the process of blood vessel formation. In bone tissue engineering, by virtue of the presence of endothelial cells, 3D-printed scaffolds can rapidly form a vascular network and integrate with the circulatory system to provide nutrients to the defect site, rapidly transport metabolic waste, and accelerate bone repair [187]. According to the 3D printing bioink composition, 3D-printed scaffolds are classified into three main categories: 3D-printed scaffolds with “cell” modification, 3D-printed scaffolds with composite cell ink, and 3D-printed scaffolds with “gene”-modified cell ink.

### 3D-Printed Scaffolds with “Cellular” Modifications

**Fig. 6:**
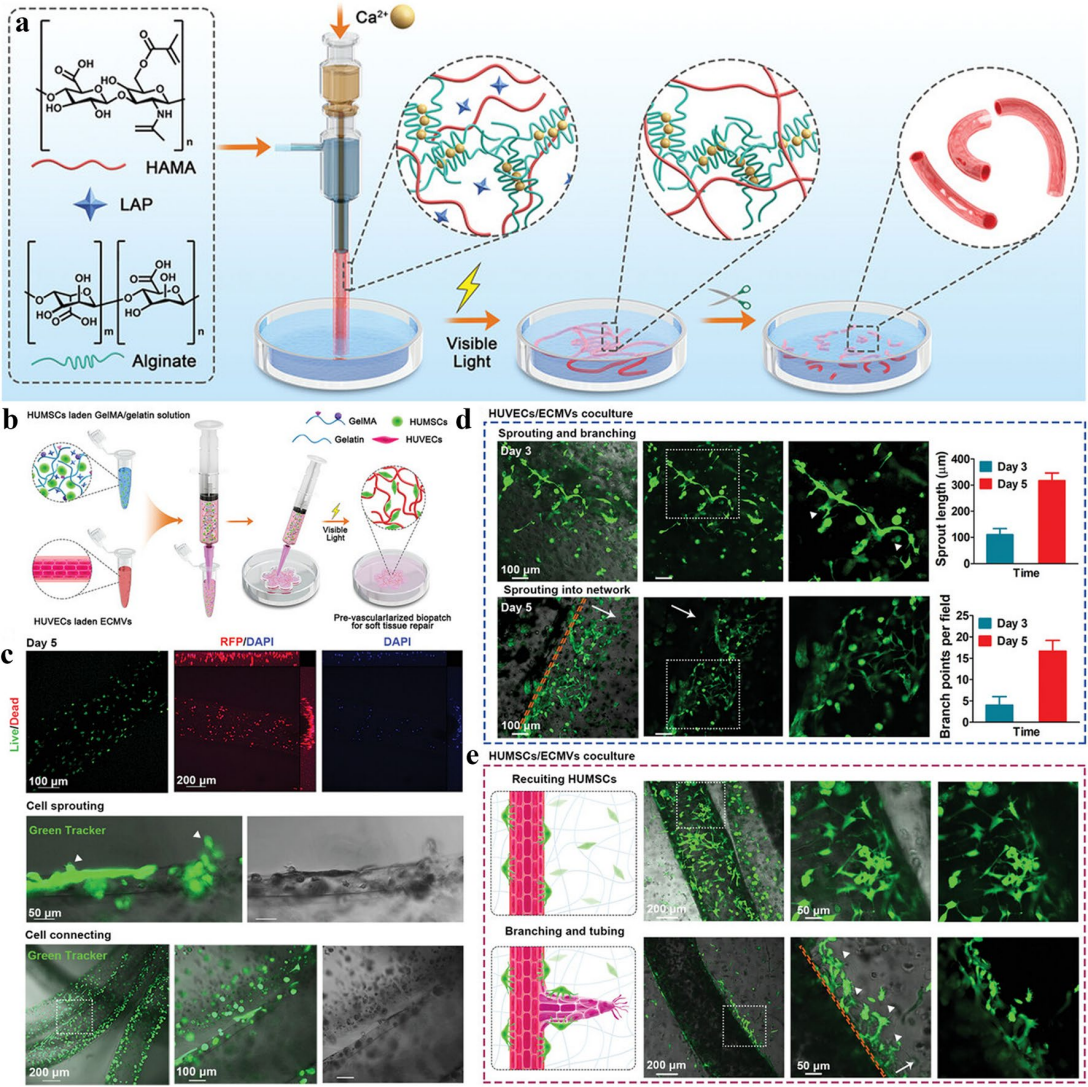
The cell-based and gene-based bioactive 3D printing vascular networks. **a** Schematic view of the printing process with cell-based bioink and microstructure images of the printed scaffolds. **b** Schematic diagram of the preparation of a repair hydrogel vascular scaffold containing human umbilical cord mesenchymal stem cells (HUMSC). **c** Live-dead staining to test the effectiveness of hydrogel vascular scaffolds for sprouting. **d, e** Confocal microscopy images tracing hydrogel vascular scaffold germination length and branching points over time. White triangular arrows indicate tip cells. While the long arrows indicate the invasion the direction of the newly formed network. **a**–**e** are reproduced with permission [190]. Copyright 2022, Wiley

Guduric et al. constructed polylactic acid (PLA) scaffolds with cell modification using layer-by-layer assembly. Therein, the PLA scaffold was prepared by 3D printing, and human bone marrow stromal cells (hBMSCs) and endothelial progenitor cells (EPCs) were then inoculated on the surface of the scaffold. The in vitro study demonstrated that this unique scaffold assembly promoted homogeneous cell colonization and proliferation within the scaffold, ensuring homogeneous distribution and adequate vascularization of cells inside the scaffold, which may have a broad range of applications in bone tissue engineering [188]. Kuss et al. proposed the concept of “pre-vascularization”, which first used 3D printing technology to prepare a polycaprolactone/hydroxyapatite composite scaffold, followed by the surface modification of hydrogels loaded with human adipose-derived mesenchymal stem cells (ADMSCs) and HUVECs as a coating of the composite scaffold. In this study, it was found that co-culturing ADMSC–HUVEC with porous 3D-printed scaffolds in vitro can generate capillary-like networks. In vivo experiments demonstrated that the hydrogel system facilitated micro-vessel and lumen formation and promoted the anastomosis of the human-derived vascular network with the host murine vascular system. These results also demonstrated the potential of “pre-vascularization” of 3D-printed scaffolds for the preparation of large bone defects [189]. Li et al. combined cell-laden bioink coaxial microfluidics and microsurgical techniques to construct bionic engineered custom microvessels with controlled length and budding type of vascularization. The microvessels have excellent injectability and sutureability and can be effectively introduced into 3D printed implants to promote tissue vascularization [190] (Fig. 6a–e).

### 3D-Printed Scaffold with Composite Cell Ink

Kuss et al. introduced the concept of short-term hypoxic preconditioning based on “pre-vascularization”. The bioink for the 3D-printed scaffold in this study was composed of polycaprolactone/hydroxyapatite (PCL/HAp) and stromal vascular fraction cells (SVFCs)-laden hydrogel. The scaffolds pretreated in normoxic or short-term hypoxic environments were subsequently implanted into athymic mice. It was found that short-term hypoxic conditions promoted ex vivo microvessel formation and accelerated the integration with existing host vessels, as well as the potential of pre-vascularization for 3D bone tissue engineering applications [191]. Chen et al. used hydrogels loaded with MSCs and ECs to prepare dopamine-modified calcium polydopamine-modified calcium silicate (PDACS)/PCL scaffolds [192]. Kang et al. fabricated a 3D printed bone construct through an integrated tissue-organ printer (ITOP), and successively enhanced mechanical stability of bioink by effectively combining cell loaded hydrogels with biodegradable polymers. In vivo experiments show that the 3D printing scaffold with composite cells has better effect of vascularization and osteogenesis without central necrosis. Von Willebrand factor (vWF) immunostaining showed large blood vessel formation within newly formed bone tissue throughout the bioprinted bone constructs, including the central portion. Evaluation of the vascularization ability of the composite scaffolds and their cellular behavior in vitro showed that this cell-rich 3D-printed scaffold not only enhanced the expression of BMP-2 but also stimulated the development of vascular networks [193]. Rukavina et al. adopted extrusion-based and drop-on-demand bioprinting techniques to construct pre-vascularized bone tissue with bioink-containing human adipose-derived mesenchymal stem cells (hASCs) and human umbilical vein endothelial cells (HUVECs). In particular, the formation of a micro-vessel network was detected in vivo experiments but also the formation of the calcified bone matrix was observed, which is important for vascularized osteogenesis [194].

### 3D-Printed Scaffold with "Genetically" Modified Cell Ink

The effective combination of genetic engineering and regenerative medicine can further enhance the functionality of vascularized scaffolds. Lin et al. constructed bone marrow mesenchymal stem cells bearing the BMP-2 gene by lentiviral transfection and subsequent introduction of the specifically functionalized stem cells into 3D-printed hydrogel scaffolds. The results showed that BMP-2-HBMSCs encapsulated in the scaffold could highly express BMP-2 in a sustained manner, and histological staining 14 days after implantation showed that BMP significantly promoted the formation of new bone and blood vessels [195]. Cunniffe et al. developed a gene-activated bioink using plasmid, alginate, BMSCs, and nano-hydroxyapatite, which was co-printed with PCL. This study demonstrated in vitro that BMSCs that were successfully transfected by plasmids sustained high expression of BMP-2 and TGF-β3, thus promoting high levels of vascularization and mineralization [196]. Pizzicannella et al. used MSC cells and extracellular vesicles (EVs) in combination with PLA to form a bioink. Although cell transfection was not involved, the extracellular vesicles could carry and transfer proteins, mRNA, and microRNA to the target cells. In the subsequent ex vivo experiments, it was evidenced that the presence of EVs significantly increased the expression of relevant genes and accelerated the vascularized regeneration of cranial bone [197].

## Bionic 3D-Printed Scaffold for Vascularization in Tissues

The concept of "bionics" is attracting more and more attention in the field of biomaterials. In 1960, bionics was formally proposed as a discipline by Steele at a bionics conference in the USA [198, 199]. Bionics is an interdisciplinary field that combines biology and technology, where the principles of structural and functional work of living organisms are studied and new devices, tools, and technologies are invented based on these principles to develop advanced technologies for production, learning, and life. It is well known that bone tissue is a highly dense connective tissue with a unique structure [200–202]. It consists mainly of peripheral cortical bone—the “shell”—and central cancellous bone—the “core”. Therefore, it is also a specific “core–shell” structure [203]. The cortical bone, also called dense bone, is composed of closely spaced bone units, maintaining the hardness and denseness of the bone [204]. There are Haversian canals in the cortical bone with abundant blood vessels and nerves in the longitudinal direction [205]. The vessels in the Haversian canals are interconnected by vessels in the transverse direction, called Volkmann’s canals. The cancellous bone is spongy and consists of intertwined trabeculae, which have abundant blood flow and can also be seen as a robust capillary network distributed throughout the interior of the bone [42, 206]. Besides, bone tissue can also be regarded as a huge network of calcium and phosphate, which are smartly integrated with blood vessels, “communicate” with each other and together maintain the dynamic balance of the bone tissue [43, 207, 208]. In the field of bone tissue engineering, “bionics” and biomaterials have been strongly linked, both in terms of bone tissue structure and composition, to more closely resemble natural bone tissue, with better biocompatibility and osseointegration capabilities [209]. Eventually, based on the great challenge of how to completely “centralize” the vascularization of bone tissue engineering scaffolds, bionic scaffolds have emerged. Bionics can be divided into three main categories: 3D-printed scaffolds (“shell”) and hydrogels (“core”), 3D-printed scaffolds (“shell”) with hollow pipes (“core”), and 3D-printed stents (“shell”) with vascular tips (“core”).

### 3D-Printed Scaffolds ("Shell") and Hydrogels ("Core")

**Fig. 7:**
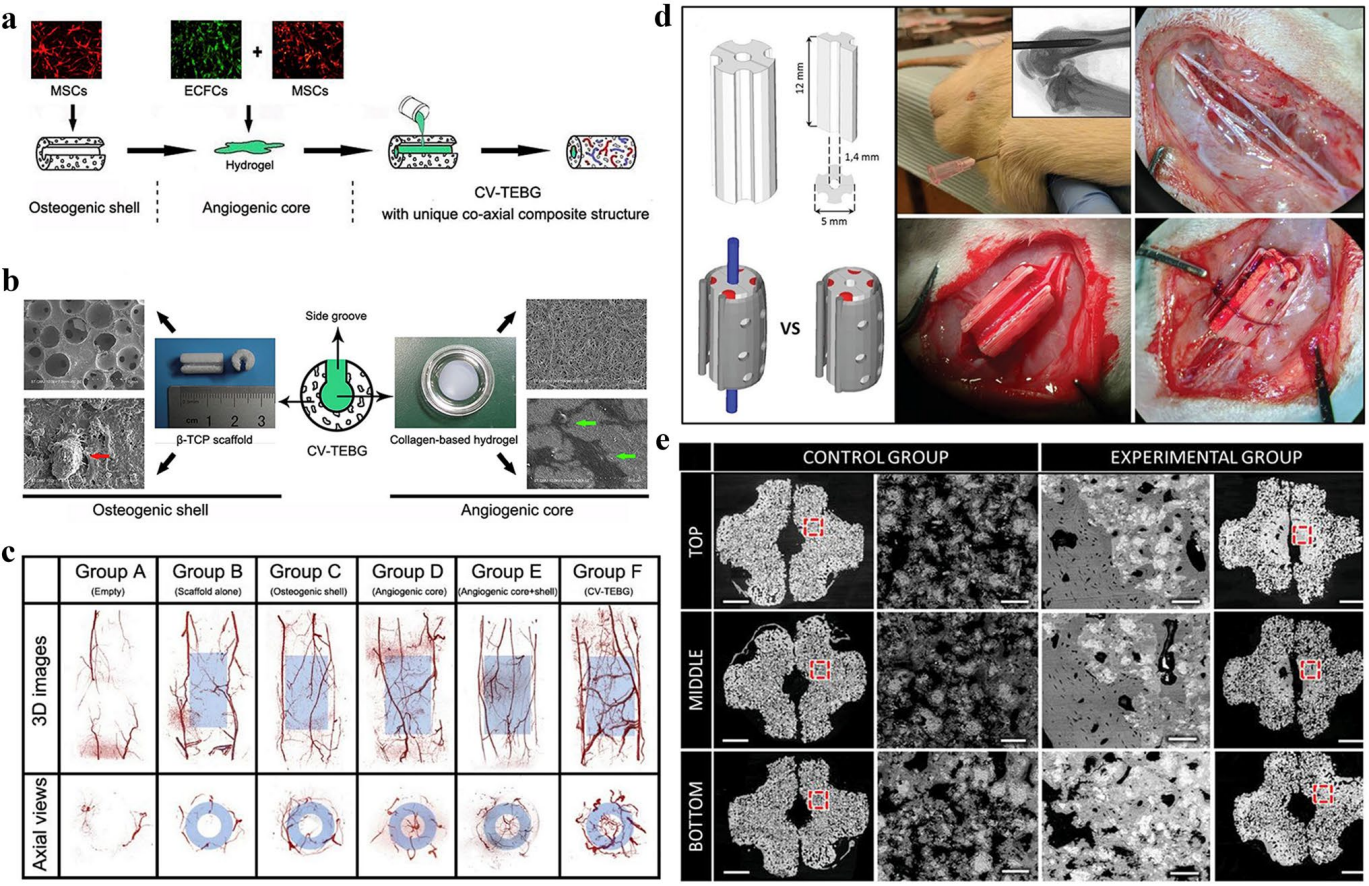
Structural mimicking 3D printing approaches. **a** Experimental design of a centrally vascularized tissue engineering bone graft with the unique core–shell composite structure. **b** SEM characterization and cellular adhesion of implanted cells to the scaffolds. **c** Evaluation of neovascularization of different types of scaffolds through enhanced micro-CT imaging in rabbit femoral bone defect model. **a**–**c** are reproduced with permission [210]. Copyright 2018, Mary Ann Liebert, Inc. **d** Marrow retaining scaffold design and dimensions and positioning of vessels with perforated plastic retainer and scaffold assembly in plastic retainer. **e** Backscattered SEM axial sections from the top, middle, and bottom of scaffolds with high magnification regions. **d**, **e** are reproduced with permission [214]. Copyright 2019, Wiley

Wang et al. proposed the concept of centrally vascularized bone tissue engineering by first printing a β-TCP scaffold through 3D printing technology and then coculture it with MSCs cells in vitro as the “shell” for osteogenesis (Fig. 7a–c). Subsequently, MSC and ECFC cells were incorporated into rat tail collagen and plasma fibronectin to fabricate a composite hydrogel, which was then injected into the 3D-printed scaffold as a vascularized “core”. This unique “core–shell” structure outperformed other osteogenic or vascularized scaffolds in vitro, demonstrating excellent osteogenic and vascularization ability. Furthermore, it formed a unique burrito-like vascular network around and in the center of the scaffold, indicating a potential synergy between the osteogenic shell and the vascularized core. This successfully bridged the defect between the outer cortical bone-like tissue and the inner cancellous bone-like tissue, revealing the importance of vascularization in osteogenesis [210]. Anada et al. utilized a two-step 3D printing technique and first printed an outer gel ring of octacalcium phosphate (OCP)—GelMA to mimic cortical bone, followed by using HUVECs spheroids and gelatin methacrylate (GelMA) hydrogel as the raw materials to print a central GelMA ring-containing HUVECs spheroids to mimic the bone marrow space. This experiment demonstrated that HUVECs spheroids formed capillary-like structures in vitro and showed that this composite scaffold could stimulate the osteogenic differentiation of MSCs, further demonstrating the association between vascularization and osteogenesis [211].

### 3D-Printed Stent ("Shell") and Vascular Tip ("Core")

Vidal et al. used 3D printing technology to print customized calcium phosphate scaffolds as the “shell” for sheep bone defect areas. They treated each of three identical groups of sheep either with blank control, calcium phosphate scaffolds alone, or calcium phosphate scaffolds with axial vascular pedicle. This study demonstrated that the presence of the pedicle further enhanced bone formation, and the histological results confirmed that the porous 3D scaffold containing the vascular pedicle can induce the most active bone growth, resulting in the best osteogenic outcome [212]. Li et al. constructed rhBMP-2-loaded chitosan microspheres by an emulsion cross-linking method and prepared a (PLGA/β-TCP) dual-component scaffold by 3D printing [213]. Charbonnier et al. first used 3D printing technology and fabricated microporous bioceramics scaffolds, loaded with autologous total bone marrow obtained by needle aspiration. The femoral vein was then inserted into the center of the stent to assess the effect of a central flow-through vein on bone formation from marrow in a subcutaneous site. In vivo experiments showed that there were a lot of new bone formation in the experimental group with intravenous perfusion. This is the first report illustrating the capacity of an intrinsic vascularization by a single vein to support ectopic bone formation from untreated marrow (Fig. 7d, e). Then, the rhBMP-2-loaded chitosan microspheres were uniformly injected into the 3D-printed scaffold and freeze-dried as an osteogenic shell structure, followed by the implantation of arteriovenous bundles in the center to prepare vascularized bone flaps. It was demonstrated that the controlled release of rhBMP2 from chitosan microspheres could successfully induce ectopic bone formation within the scaffold. In addition, owing to the presence of central arteriovenous bundles and the coupling of osteogenesis and vascularization, this composite scaffold significantly accelerates the osteogenic process [214].

### 3D-Printed Scaffold ("Shell") and Hollow Pipe ("Core")

**Fig. 8:**
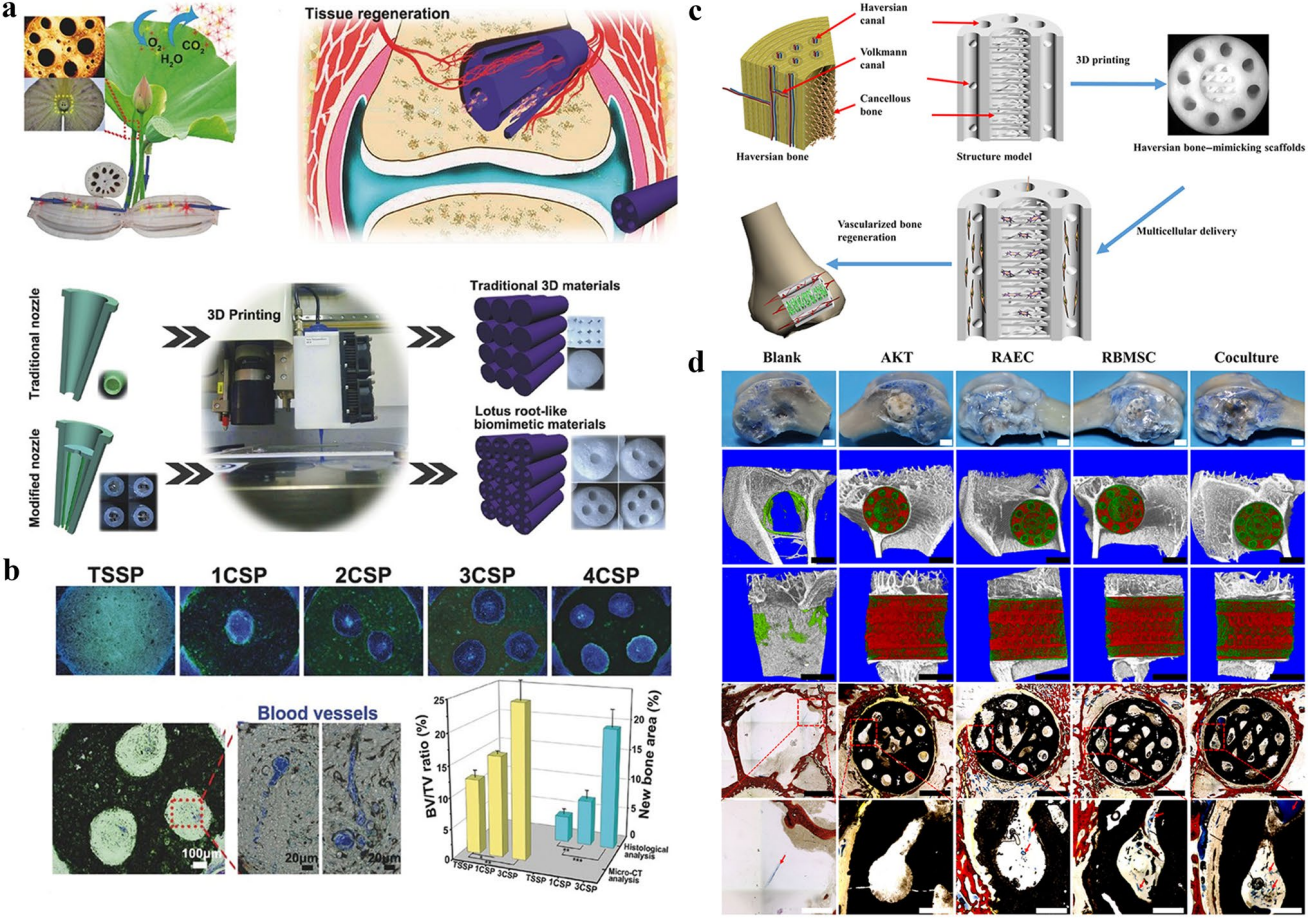
Structural mimicking designs for vascularized tissue constructs. **a** The schemata of the functions of lotus root microstructure and the application of lotus root-like biomimetic materials in tissue regeneration. Traditional and embedded structure of 3D printed nozzle and the printing process. **b** Fluorescence image of histological sections of biomimetic scaffolds, the optical and magnified blood vessels within the scaffolds, and the micro-CT analysis of newly formed **c**, bone tissue. **a**, **b** are reproduced with permission [215]. Copyright 2017, Wiley. **c** Bioprinting process of the Haversian bone-mimicking scaffolds. **d** The histological studies of the Haversian bone-mimicking scaffold implants to evaluate vasculogenesis and osteogenesis properties. **c**, **d** are reproduced with permission [217]. Copyright 2020, American Association for the Advancement of Science

Inspired by the "lotus root", Feng et al. fabricated bionic hollow pipe structures, which are an ideal model with low density, high porosity, and low flow resistance (Fig. 8a, b). With the hollow and porous scaffolds prepared by 3D printing, the porous structures can promote blood perfusion, increase the transportation efficiency of oxygen/nutrients and metabolic wastes, and induce tissue growth along the porous channels. This study demonstrated that the bionic "lotus root" 3D-printed scaffolds can significantly enhance cell attachment and proliferation in vitro and promote vascularization and osteogenesis in vivo [215]. Zhang et al. adopted the coaxial 3D printing technique with a modified core/shell printer nozzle and a viscoelastic bioceramic slurry to produce a silicate bioceramic (BRT–H) scaffold with an outer diameter of 1 mm and an inner diameter of 500 μm. The compressive strength of the scaffold was up to 26 MPa. The BRT scaffold cannot only affect osteogenesis by releasing bioactive ions but also promote angiogenesis by inducing endothelial cell migration. More interestingly, the hollow pipes can significantly facilitate the host vessels permeate into the pipes and of great benefit for transporting stem cells and growth factors to promote vascularized osteogenesis [216]. Based on digital laser processing (DLP), Zhang et al. successfully prepared a Haversian bone-mimicking scaffold with complete hierarchical Haversian bone structures. By altering the parameters of the Haversian bone-mimicking structure, the compressive strength and porosity of the 3D-printed scaffold could be manipulated. The Haversian bone-mimicking scaffold showed great potential for multicellular delivery, as it could induce osteogenesis, angiogenesis, and neurogenic differentiation in vitro, and boost the ingrowth of vessels and the formation of new bone in vivo. By mimicking native complex bone tissue, this study provides a novel strategy to design biomaterials that are structured and functionalized for application in tissue regeneration (Fig. 8c, d) [217]. To sum up, the unique “core–shell” bionic scaffold has emerged as a solution in bone tissue engineering, particularly for the treatment of large bone defects. The superiorities in central vascularization, nutrient, and metabolic waste transportation, and the coupling of vascularization and osteogenesis endow bionic scaffolds with broad and promising application prospects.

## Vascularized 4D Printed Scaffolds in the Tissues

The main principle of 4D printing technology is based on 3D printing, using 3D printing method and the intelligent materials (SMP is the most widely used) as printing ink, to design the material functionality, molding process and final programming behavior into the initial configuration [218]. After the printing is formed, the external stimulus is used to precisely control the change of the shape and functional of the 4D printing objects to meet the needs of the application [219]. The external stimulus mainly includes physical factors (temperature and humidity, force, sound and photoelectricity, time), chemical factors (pH, acid and base, catalyst) and biological factors (sugar salinity, enzyme) [220]. These changes mainly include physical changes (geometry, specific gravity, structure, force, light, electric, thermal and magnetic properties), chemical changes (oxidation, reduction, catalysis and stability) and physiological changes (cell activity, enzyme activity, reproduction and repair), which changes are usually spontaneous, change over time, do not need own energy, and can use energy stored or transformed from external conditions and innovative technologies that integrate product design, manufacturing, and assembly have been realized [221]. The 4D objects are no longer static and inanimate, but intelligent and dynamic. The material can be directly “programmed” to design the deformation of the structure into the material and simplify the manufacturing process. In conclusion, 4D printing technology is an additive manufacturing-technology of intelligent materials.

Smart materials, also known as intelligent materials, which are responsive to the external stimulus including heat, moisture, stress, pH, and magnetic fields, have found extensive applications in sensors, actuators, soft robots, medical devices and artificial muscles, which mainly includes Shape memory polymer (SMP), shape memory alloy (SMA), shape memory hydrogels (SMHs), electroactive polymer (EAP) and etc. [222]. Shape memory polymer is the most widely used intelligent material in the field of 4D printing. It refers to a kind of polymer material that can change their shape and functional according to the external stimulus and adjust its state parameters, so as to back to the preset state [223]. Compared with shape memory alloy, it has better plasticity, good biocompatibility, mechanical properties and shape memory properties. Shape memory alloy is an intelligent material which can be used to restore shape by thermal drive [224]. It has the characteristics of low driving voltage, high strain performance, large rigidity and wide strain range, and has great value in the application of 4D printing technology. Shape memory hydrogel (SMHs) is also a very popular intelligent material [225]. Its working mechanism is that the molecular chains in polymers undergo reversible hydration under different conditions and change volume through shrinkage and swelling so as to realize the shape memory function of SMHs [226, 227]. The shape memory hydrogel is mainly driven by water. The printed structure has a great dependence on the environment. To achieve the shape change without changing the volume of the hydrogel, more complex chemical reaction and structural design are needed. 4D printing technology is one of the methods to change the shape of SMHS, which has been applied in the medical field [228]. Electroactive polymer (EAP) is a new type of intelligent material which can produce shape change under electric field excitation. It has special electrical and mechanical properties. Compared with traditional piezoelectric materials, EAP has the advantages of light weight, high driving efficiency and good seismic performance. The artificial muscle actuator based on EAP combined with 4D printing has greater strain performance, which is one of the most potential biomimetic materials [224].

**Fig. 9:**
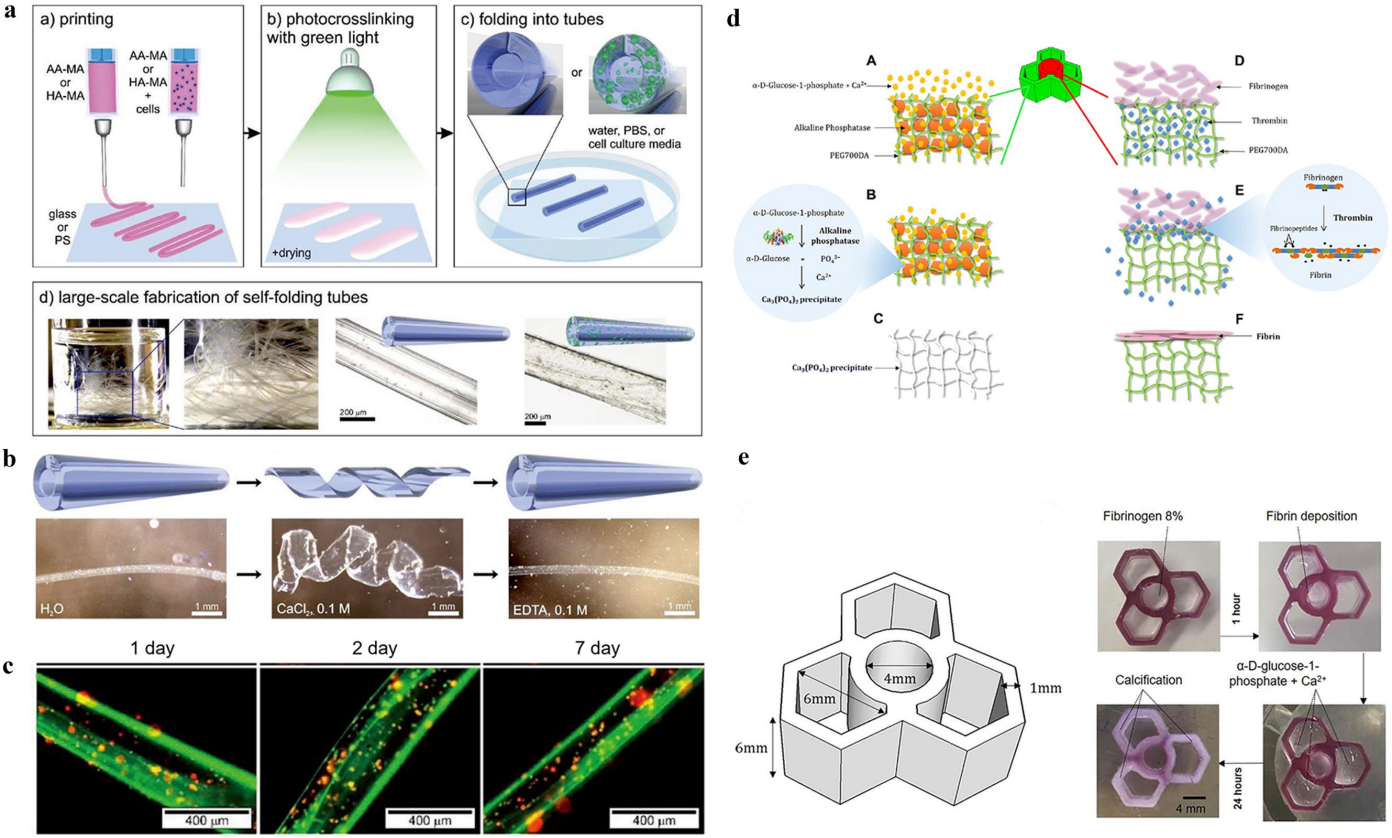
Schematic illustration of the 4D printed vascularized scaffolds application in tissue engineering.** a** Scheme of the 4D biofabrication of self-folding hydrogel-based (cell-laden) tubes. **b** Self-folding hydrogel-based (cell-laden) tubes responsiveness: cartoons (upper panel) and representative photographs (lower panel). **c** Representative fluorescence microscopy images of the cell-laden tubes after 1 d (left column), 2 d (middle column), and 7 d (right column) of culture [227]. Copyright 2017, Wiley. **d** Alkaline phosphatase and thrombin 4D activity [225]. Copyright 2018, Wiley. **e** The sequential catalysis activities of 4D object leading to fibrin deposition and calcification [225] Copyright 2018, Wiley

Bone tissue regeneration is a dynamic process, and these dynamic functional conformation changes are caused by internal mechanisms that respond to internal or external stimuli, which cannot be simulated by 3D printing [229]. The 4D printed objects can change over time under different stimuli and adapt to the native microenvironment of the defect area, providing a new strategy for bone tissue engineering [228, 230]. In addition, the biomimetic bone microenvironment established by 4D printing also affects the cell behavior in the post-printing stage and enhances the differentiation of stem cells [224]. In vascularized bone tissue engineering, the rapid establishment of a microvascular network is the key to success. The diameters of natural capillaries and venules are mostly 8–20 μm. The cortical bone also contains the Haversian canal, in which the diameter of the central vessel is 60–90 μm. Traditional 3D printing technology is unable to meet the requirements. Based on the shortcomings of 3D printing, Kirillova et al. used 4D printing technology to be fabricated hollow self-folding tubes with diameters comparable to those of the smallest blood vessels by combining mouse MSCs with methacrylate alginate and hyaluronic acid hybrid hydrogels [227]. This kind of blood vessel can maintain the cells in the hydrogel to survive for at least 7 days in vitro, and the cell viability is not affected. The successful preparation of such microvessels is of great significance to vascularized bone tissue engineering (Fig. 9a–d). In another study, Devillard et al. used 4D printing to embed thrombin and alkaline phosphatase in bio-ink [225]. The encapsulated alkaline phosphatase can make local and pre-programmed calcifications of 3D object parts. And the diffusion of thrombin from the object allows the formation of fibrin biofilm directly on the surface of the 3D object. Through this special method, it can make vascularized alveolar bone constructs. This research also provides a new direction for the manufacture of multi-active 4D printed vascularized bone tissue (Fig. 9d, e). These advances in 4D printing technology can modify the traditional 3D printed bone structure, enhance its shape and function adaptability, and provide additional potential for manufacturing complex printed bone structures in future clinical applications to dynamically adapt to defective areas.

## 5D/6D Printing in Bone Tissue Engineering and the Challenges of Industrialization

The printing technology revolution is underway, with 5D and 6D printing developing as two emerging innovative and widely used technologies, while 3D and 4D printing are facing challenges. The 5D printing technology was first implemented by William Yerazunis, Senior Principal Research Scientist at Mitsubishi Electric Research Labs (MERL), in 2018 [231]. Besides, professor William Yerazunis made the initiative to step into the future and potentially take 5D printing to new heights. In contrast to 3D printing technology, 5D printing can be done on the traditional X, Y and Z axes, with the addition of two other axes, which involves the motion of the printing head along with the rotation of the print bed at defined angles [232]. 5D printing is an emerging additive manufacturing-technology that can create multi-dimensional products especially curved layers using five-axis printers whose printer heads & printable objects have five degrees of freedom as the printer bed can move back and forth with additional two axes. The competitive advantage of 5D printing compared with 3D printing is that it can produce stronger objects using less material. As the human skeleton is not a simple flat surface but a curved surface with a certain degree of curvature, 5D printing can meet the needs of this artificial bone, providing strong mechanical properties while having a curved surface, 5D printing has great potential for artificial bone production [233, 234]. In Orthopedic surgery, complex and strong implants with curved surfaces are an essential requirement. 5D printing prints these complex surgical implants as per actual surgery of patient and also applicable in surgical planning, teaching, and learning. So, 5D printing can easily create a sophisticated and curved structure which requires a lot of strength [15, 234]. Here, an attempt is made to introduce the concept of 6D printing, which can be regarded as a child born from the marriage between 4D printing and 5D printing. The main advantage of 6D printing is the ability to print in more directions and along more complex and even curved paths, thus leading to more elaborate products in terms of achieving a level of structural integrity and intelligence. In addition, a properly set up approach to 6D printing can reduce the use of raw materials while providing shorter processing times due to its inherent processing flexibility [16]. Note that, on the whole, the proposed idea aims to improve the quality, programmability and tunability of the multi-intelligent response of printed objects to meet the needs of vascularized bone tissue engineering. Apart from the advantages mentioned above, the main limitation of 5D and 6D printing in terms of industrialization is the additional cost of the two axes, which people do not value as a technological revolution. The 5D and 6D printing covered in this overview refers to the actual degrees of freedom of the print head and print platform in the printing device. Furthermore, it is worth noting that printers with 5D and 6D degrees of freedom still perform 3D/4D printing. Running bespoke machines also requires more accurate software and hardware, which is a challenge for both individuals and society. Another limitation is the highly skilled human resources required to operate and maintain the machines, which means more expenditure in terms of time and money.

## Vascularized Additive Manufacturing-Scaffolds in General Tissue Engineering Fields

**Scheme 2:**
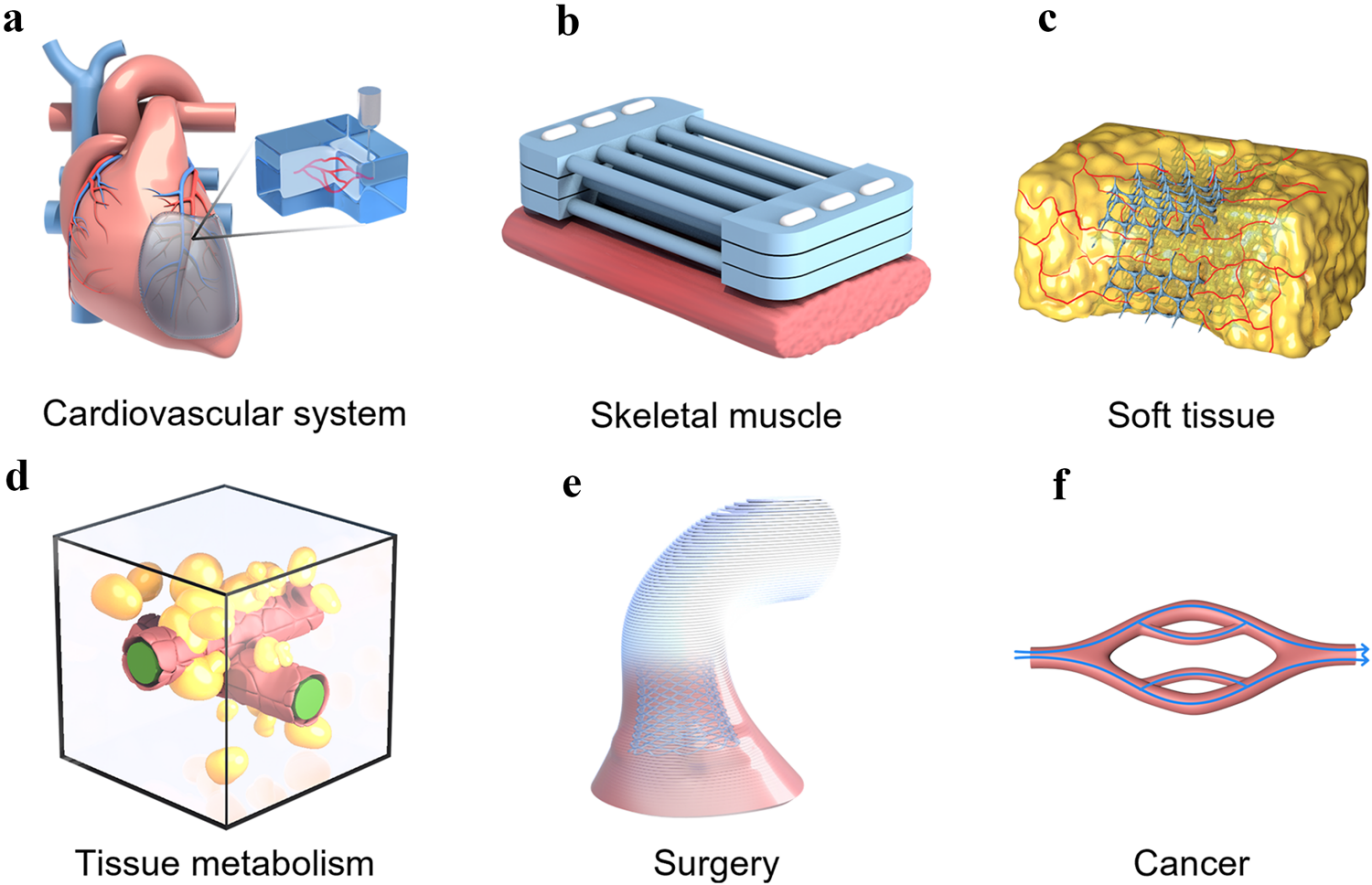
Schematic illustration of the vascularized additive manufacturing-scaffolds application in related areas. **a** Cardiac patch with unique 4D self-deforming capability [248]. **b** Biomimetic muscle fiber structure of 3D printed skeletal muscle [193]. **c** Self-filled vascularized soft tissue [250]. **d** Vascularized 3D printed objects with self-generated oxygen [252]. **e** Patient-specific aortic root models with internal sensors [253]. **f** Endothelialized vascular beds to elucidate the contribution of hydrodynamics in determining sites of circulating tumor cell (CTC) vascular colonization [254]

Tissue engineering has been a brand-new approach to solve the problems of tissue repairing and organ reconstruction in regenerative medicine [23, 235, 236]. Additive manufacturing-technology can construct complex and systematic vascular networks, then protect and mimic the metabolic functions of natural tissues, and thus provide the possibility for further applications of tissue engineering [8]. In this paper, the application of additive manufacturing-technology in vascularized bone tissue engineering has been reviewed. Furthermore, the application of additive manufacturing-technology in vascular tissue engineering, cardiovascular system, skeletal muscle, soft tissue (adipose tissue and skin), tissue metabolism, surgery and cancer in this chapter is briefly presented (Scheme 2). The construction of functional vascular networks not only enables tissue repair and regeneration, but also facilitates research and development of tumor therapy and artificial organs, which is of great significance for improving human health. Vascularization engineering is widely used because it can be constructed through different technical routes, greatly increasing the operability and control of vascularization engineering [237]. There are a number of methods for constructing vascularized tissue engineering, including sound Patterning, volumetric printing, guidance provided by anisotropic particles and gels, DLP printing, among others. (1) Sound Patterning involves the use of acoustic surface standing waves (e.g., Faraday waves) to drive cells, organoids or inorganic particles to form a target spatial pattern on demand. The technique allows parameters (sound frequency, amplitude, chamber shape) to be adjusted to generate morphologically relevant tissue under non-contact, rapid and gentle culture conditions. Both Petta et al. and Marzio et al. have been successful in studies of acoustically induced angiogenesis [238, 239]. (2) Volumetric printing, an ultra-fast, light-based technology that sculpts cell-filled hydrogel bioresins into 3D structures in a layer-less manner, allows for spatial patterning of multiple ink/cell types, offering greater design freedom than traditional bioprinting. Größbacher et al., Ribezzi et al., Falandt et al. and Bernal et al. have all achieved success in vascularisation engineering using bio-volume printing, even with large vessels and microcapillaries [240–243]. (3) Licht et al., Terpstra et al. and Tang et al. have been successful in the field of vascularization engineering using guidance provided by anisotropic particles and gels, DLP printing, in acute spinal cord injury, meniscal models and tumor models respectively [244–246]

The contractile activity of the heart is regarded as the engine of the cardiovascular system, in which the blood vessels have a complex tree-like structure, including the smallest capillary with a diameter from 5 to 10 mm and the largest artery with a diameter of 30 mm [5]. With the development of 3D/4D vascular network, the use of 3D/4D printed technology to reconstruct vascular tissue has been the focus of clinical research [247]. Scott et al. reported a biological manufacturing method, via embedded three-dimensional bioprinting, which introduces vascular networks into organ building blocks (OBBs), and the matrix shows the required self-healing and viscoplastic behavior, and the subsequent construction of perfusable cardiac tissue with synchronously fusing and beating activity [27]. In another study, this specific design improves the biomechanical properties of the patch itself and the dynamic fusion of the patch with the beating heart by combining the unique 4D self-deforming capability with an expandable microstructure. Vascular and myocyte maturation were improved in vivo and in vitro under physiologically related mechanical stimulation [248] (Scheme 2a). Skeletal muscle is connected to the bone through tendons, and the movement is controlled by the force generated by contraction. In addition, skeletal muscle is also a kind of high vascularized tissue as bone. In order to achieve effective transmission, vascularized skeletal muscle printing needs to control the vascular network more accurately. Kang et al. presented an integrated tissue–organ printer (ITOP) and fabricated an organized soft tissue—a 3D muscle construct 15 mm × 5 mm × 1 mm in dimension. Immediately after printing, the printed structures contained muscle fiber–like bundles (~ 400 µm width), supporting PCL pillars and Pluronic F-127 hydrogel as a temporary structure [193] (Scheme 2b). Soft tissues include skin, adipose tissue, nerves, etc. Of these, the skin is the largest organ of the human body and fatty tissue is also involved, covers the surface of the body, providing protection and indispensable sensitive such as feeling, secretion, excretion, breathing and other functions [249]. The soft, elastomeric aliphatic polycarbonate-based materials were designed by Weems et al. to undergo 4D printing into supportive soft tissue engineering scaffolds. The 4D printed scaffold allowed them to fill model soft tissue voids without deforming the surrounding material, and in vivo, infiltration of adipocyte lobules into surface eroded scaffolds can be observed at 2 months, while neovascularization is observed [250] (Scheme 2c). In addition to applications in macroscopic tissues, there are also applications in microscopic cellular metabolism. Miller et al. printed rigid 3D filament networks of carbohydrate glass that could be lined with endothelial cells and perfused with blood under high-pressure pulsatile flow. The perfusion of vascular channels maintained the metabolic function of primary rat hepatocytes, whose core function would otherwise be inhibited [251]. In another study, Zhang et al. at Harvard University, used three-dimensional (3D) bioprinting containing patterns of green algae (Chlamydomonas reinhardtii) as a natural photosynthetic oxygen generator. This enabled photosynthetic oxygen (O_2_) to be supplied to the volumetric extracellular matrix within mammalian cells. The researchers were able to enzymatically remove the pattern of bio-printed Chlamydomonas reinhardtii from the matrix after subsequent reendothelialization of the formed hollow perfusable microchannels, resulting in biologically relevant vascularized mammalian tissue constructs [252] (Scheme 2d). What's more, It is also used in clinical treatment, mainly in surgery and oncology. Haghiashtiani et al. printed patient-specific aortic root models with internal sensors for minimally invasive applications. These models may pave exciting avenues for mitigating the risks of postoperative complications and facilitating the development of next-generation medical devices [253] (Scheme 2e). In another study, Gillaspie et al. used 5D printing technology to develop models of complex tumors, which helped with surgical planning, predicting potential difficulties, reducing surgical risks, teaching learners and educating patients [15]. Hynes et al. have bio-printed endothelialized vascular beds and perfused these constructs with metastatic mammary gland cells under physiological flow rates to elucidate the contribution of hydrodynamics in determining sites of circulating tumor cell (CTC) vascular colonization [254] (Scheme 2f). All kinds of application examples show that additive manufacturing has a broad application prospect in the field of regenerative medicine and provides a cutting-edge method for the structural design of organ regeneration complex tissues. By now, the additive manufacturing of some tissues and organs that have been completed is still far from clinical application [5, 255–257].How to improve the mechanical strength while building a blood vessel network in a shorter time to promote tissue survival and regeneration is the main direction of future efforts.

## Conclusions and Prospects

Timely and effective vascularization has always been a key to bone repair. Additive manufacturing-technology can effectively combine tissue engineering and vascularized osteogenesis and print multi-functional scaffolds for clinical transplantation [67]. It has become an emerging, precise, and personalized technique for constructing vascularized bone tissue engineering scaffolds. Although several reviews have discussed 3D/4D printing scaffold vascularization, to the best of our knowledge, this review is the first comprehensive review of vascularized 3D/4D/5D/6D-printed bone repair biomaterials that includes research advances in vascularization in tissue repair and regeneration, tissue graft materials, techniques, and materials of 3D printing, 3D-printed scaffolds for vascularized osteogenesis, 4D printing, materials and applications in vascularized bone tissue engineering, the excellent advantages of 5D/6D printing scaffolds in bone tissue engineering and vascularized additive manufacturing-scaffolds in related fields, Even though many successful cases of vascularized additive manufacturing-scaffolds have been reported, there are still several challenges that need to be focused on and overcome.

1. Different regions of human tissues may have various biomechanical properties and structural architectures, and the complex microenvironment of tissues makes bionic printing more difficult [206]. Therefore, obtaining precise data to print personalized vascularized scaffolds and further improving the osseointegration ability of the scaffolds will be a focus of future research.
2. For tissue functionalization and carrier loading of 3D/4D/5D/6D-printed vascularized scaffolds with bioactive small molecules, the biggest limitation is that the release behavior is not sufficiently sustained during the whole tissue repair process. Evaluation of the results showed that the most sustained release was only about four weeks, which is far from sufficient for the whole tissue repair process [157]. Thus, sustaining the release of active molecules with the premise of biosafety and early vascularization is the direction for future investigation.
3. Currently, the approaches to construct vascularized 3D/4D/5D/6D-printed scaffolds for tissue repair still focus on the delivery of functional groups, cells, or bioactive small molecules. Maintaining the activity of the bioactive components that modified the scaffold surface or were encapsulated inside the scaffold and ensuring a sustained release are needed to be explored and investigated in future studies [258–262].
4. As a strong connective-tissue that covering the tissue surface periosteum is important for tissue repair because it provides the blood supply and nutrients for tissue repair. In addition, the periosteum is abundant in nerves, thus tissue fracture also incurs nerve damage [263, 264]. Investigating the combination of vascularized additive manufacturing-tissue scaffolds and nerve regeneration is believed to further promote the research on tissue repair biomaterials.
5. Tissues are in a dynamic equilibrium between the osteoclasts formed by the differentiation of hematopoietic stem cells resulting from the resorption of dead tissue and the osteoblasts derived from differentiation of mesenchymal stem cells [43]. Given that seeking a new balance between osteoblasts and osteoclasts in the application of additive manufacturing for vascularized osteogenesis also needs to be further explored.
6. Tissue mineralization is dependent on calcium and phosphorus metabolism, of which calcium ions are important for the coagulation system. When a fracture occurs, calcium ions are involved in both the blood clot formation at the early stage and affect profoundly calcium and phosphorus metabolism at a later stage [265–268]. Using additive manufacturing-technology to alter vascularized osteogenesis by regulating calcium ions would be a valuable research direction.
7. The immune microenvironment has been shown to play an important role in the tissue repair process. The immune system, skeletal system, and vascular system are closely associated and share many cytokines related to the regulation of the immune microenvironment, which together maintain the stability of the tissue microenvironment [269–271]. Therefore, in the future, combining the immune system regulation with vascularized additive manufacturing-scaffolds to induce osteogenesis could be a new research topic.
8. Although 4D/6D printing integrates the concept of time, the deformation process of 4D/6D printing structure is only a simple shape change, which cannot meet the complex and changeable clinical applications [272]. What’s more, it is a challenge for us to accurately control the changes of 4D/6D printed objects while improving the printing resolution, whether the structural changes can bring about functional optimization.
9. The deformation mechanism is triggered by a variety of stimuli, such as temperature, pH, ultraviolet light and so on. When these stimulating factors change dramatically (e.g., temperature 4–40 °C, pH too acidic or too alkaline), whether they will affect the cell viability, and then affect the next tissue repair process, is what we need to tackle in the future research.
10. Although a large number of stimulus-responsive biomaterials have been used in 4D printing technology, it is very important to introduce computer design technology and complex multiple stimulus–response programs to realize the manufacture of complex self-transforming objects in order to promote the large-scale production and fine program control of 4D bio-printing in tissue engineering. However, the cost of these stimulus responses and programmable biomaterials can be expensive. At the same time, large-scale production should also be feasible and controllable. Therefore, there is still a trade-off between the feasibility of manufacturing and the advantages of 4D bio-printing.
11. As for 5D and 6D printing, it is important to find a balance between print head print angle and extrusion speed, so that each part works well together to print complex objects and keep the printed object active. This is the key to popularizing 5D and 6D printing technologies in the future.
12. Current approaches for vascularized additive manufacturing-scaffolds are mostly limited to single-factor analysis and lack the investigation of biological systems. As a result, the biological study of additive manufacturing-materials has not been fully developed yet. At the same time, the biological effects are codetermined by the tissue microenvironment and multi-system cells [272–274]. As the tissue microenvironment is in a dynamic equilibrium, it will be challenging to maintain the balance among the scaffolds’ mechanical properties, vascularized active components, biocompatibility, and implant microenvironment using additive manufacturing-technology. Finally, it is believed that our unremitting efforts and research will lead to significant advancements in the field of tissue regeneration.
13. In vitro modelling is one of the important applications of vascularized additive manufacturing-scaffolds, as it increases the degree of simulation of the scaffold and thus allows for a better study of the interaction between vascularized additive manufacturing-scaffolds and human physiology. In the study of some disease treatment processes, such as myocardial infarction and glandular duct syndrome, in vitro models can also be used as simulation objects to easily detect and predict disease progression from dynamic simulations within the blood vessels.
14. In vitro models of vascularized additive manufacturing-scaffolds can of course also provide better support for drug screening. As vascularized additive manufacturing-scaffold in vitro models are able to simulate the human vascular system, drugs often better mimic the actual situation when interacting with the scaffold models, thus improving the accuracy of drug studies.
15. Vascularized additive manufacturing-scaffolds can also be used for organ transplantation. Organ transplantation has been a popular area of research in medicine due to the lack of organs available for transplantation. In contrast, vascularized additive manufacturing-scaffolds can not only provide multiple components for cell growth in organs, but can also be precisely tailored to complex biological structures, thus providing new ideas and opportunities for organ transplantation.
